# Autophagic mechanisms in longevity intervention: role of natural active compounds

**DOI:** 10.1017/erm.2023.5

**Published:** 2023-03-30

**Authors:** Kevser Taban Akça, İlknur Çınar Ayan, Sümeyra Çetinkaya, Ece Miser Salihoğlu, İpek Süntar

**Affiliations:** 1 Department of Pharmacognosy, Faculty of Pharmacy, Gazi University, Ankara, Türkiye; 2 Department of Medical Biology, Medical Faculty, Necmettin Erbakan University, Meram, Konya, Türkiye; 3 Biotechnology Research Center of Ministry of Agriculture and Forestry, Yenimahalle, Ankara, Türkiye; 4 Biochemistry Department, Faculty of Pharmacy, Gazi University, Ankara, Türkiye

**Keywords:** Autophagy, cancer, diabetes, longevity, natural compounds, neurology, obesity

## Abstract

The term ‘autophagy’ literally translates to ‘self-eating’ and alterations to autophagy have been identified as one of the several molecular changes that occur with aging in a variety of species. Autophagy and aging, have a complicated and multifaceted relationship that has recently come to light thanks to breakthroughs in our understanding of the various substrates of autophagy on tissue homoeostasis. Several studies have been conducted to reveal the relationship between autophagy and age-related diseases. The present review looks at a few new aspects of autophagy and speculates on how they might be connected to both aging and the onset and progression of disease. Additionally, we go over the most recent preclinical data supporting the use of autophagy modulators as age-related illnesses including cancer, cardiovascular and neurodegenerative diseases, and metabolic dysfunction. It is crucial to discover important targets in the autophagy pathway in order to create innovative therapies that effectively target autophagy. Natural products have pharmacological properties that can be therapeutically advantageous for the treatment of several diseases and they also serve as valuable sources of inspiration for the development of possible new small-molecule drugs. Indeed, recent scientific studies have shown that several natural products including alkaloids, terpenoids, steroids, and phenolics, have the ability to alter a number of important autophagic signalling pathways and exert therapeutic effects, thus, a wide range of potential targets in various stages of autophagy have been discovered. In this review, we summarised the naturally occurring active compounds that may control the autophagic signalling pathways.

## Introduction

Aging is a biological process that causes the organism's quality of life to diminish over time due to time-dependent cellular and functional degradation. Age-related conditions as a group pose a serious socioeconomic burden on the world and a substantial healthcare concern. Finding treatment approaches that slow the deterioration of numerous age-related pathological disorders is therefore crucial (Ref. 1).

The incidence of various diseases such as cancer, cardiovascular diseases, neurodegenerative diseases (NDs) and metabolic dysfunction rises with aging (Ref. 2). Above-mentioned illnesses can be brought on by dysregulation of cellular autophagy. Autophagy is a quite preserved catabolic cellular process that expresses lysosomal degradation and recycling of nucleic acids, proteins, lipids and other intracellular components. Autophagy mechanism is associated with homoeostasis, differentiation, development and survival processes, as well as defence against pathogens and conservation of cellular energy. Decreased autophagic activity in aging affects a variety of cellular and molecular processes and results in the accumulation of damaged macromolecules and organelles. Therefore, it has been suggested that maintenance of regular autophagic activity is associated with longevity (Ref. 3).

Autophagy functions as both a tumour suppressor and promoter (through protective autophagy) in cancer. Furthermore, it plays a crucial role in preventing cardiac aging and maintaining neuronal homoeostasis. Early regulation of the formation of the mammalian heart, proper maintenance of cardiac structure and function, and the beginning and progression of cardiovascular disorders are all regulated by autophagic activity (Ref. 4). In addition to protein aggregation, NDs have dysregulated autophagy along with mitochondrial dysfunction and reduced lysosomal activity. The prevalence of NDs like Alzheimer's disease (AD), Parkinson's disease (PD), and Huntington's disease (HD) increases with aging and neuronal autophagy dysregulation (Ref. 5). Moreover, in metabolic illnesses including obesity and diabetes, autophagy is suppressed or increased (Ref. 6). Studies have demonstrated the significance of the signalling pathways PI3K/AKT/mTOR, AMP-activated protein kinase (AMPK), MAPK, SIRT1, and FoXO in the control of autophagy, cell aging, and metabolic diseases.

There are many synthetic compounds that have been discovered that modify autophagy and are good candidates for the therapy of cancer, but they come with unfavourable side effects. As a result, several phytochemicals have drawn a lot of interest for their potential to be used as autophagy modulators with minimum adverse effects (Ref. 7). Indeed, naturally occurring compounds including, alkaloids, terpenoids, flavonoids, phenylpropanoids, lignans, phenolic acids, stilbenes, tannins, senevol glycosides, steroids, lectins have been found to be powerful autophagy inducers or inhibitors, opening up new treatment regimens.

In the present review new aspects of autophagy and speculations on how they might be connected to both aging and the onset and progression of age-related diseases are discussed. We also aim to summarise the natural compounds that can be used as autophagy modulators. Eventually, developing practical applications that support long-term health will be made easier with a better understanding of the specific interactions between autophagy and the risk of age-related diseases across organisms.

## Autophagy and aging

Three different types of autophagy have been described; macroautophagy; is the basic mechanism of autophagy and occurs when larger particles and organelles are taken into the autophagosome and fused with lysosomes, microautophagy; small particles are taken into the lysosome with the indentation in the membrane and degraded, chaperone-mediated autophagy (CMA); the recognition and transport of cytosolic proteins with a certain peptide sequence by Hsp73, which is complex with molecular chaperones, to lysosomes and this type of degradation pathway does not require vesicular traffic (Refs 8, 9). On the other hand, selective forms of macroautophagy and microautophagy in which the degradation process occurs due to either the binding of HSC70 to the hydrophobic residues exposed in misfolded or aggregated proteins exists, namely chaperone-assisted selective autophagy (CASA) or the sequence-mediated targeting of proteins by HSC70, namely endosomal microautophagy (eMI) (Refs 10, 11).

The morphology of autophagy was first described in mammalian cells in the 1950s, and 31 autophagy-related (ATG) genes involved in the basic mechanism in autophagy were identified (Refs 12, 13). Most yeast genes encoding autophagy proteins are orthologous to the *Caenorhabditis elegans* genome (ATG2, ATG3, ATG4, ATG5, ATG7, ATG9, ATG10, ATG12, ATG18 genes), but there are two homologues of ATG4, ATG8 and ATG16. The ATG proteins encoded by these genes act at different stages of autophagosome biogenesis such as initiation of autophagy (unc-51/ATG1), vesicle nucleation (bec-1/ATG6, vps-34/VPS34), protein conjugation system (ATG-7/M7.5/ATG7, lgg-1/ATG8, lgg-3/ATG12), uptake and vesicle transformation (ATG-18/F41E6.13/ATG18) (Ref. 14).

**Figure 1.**
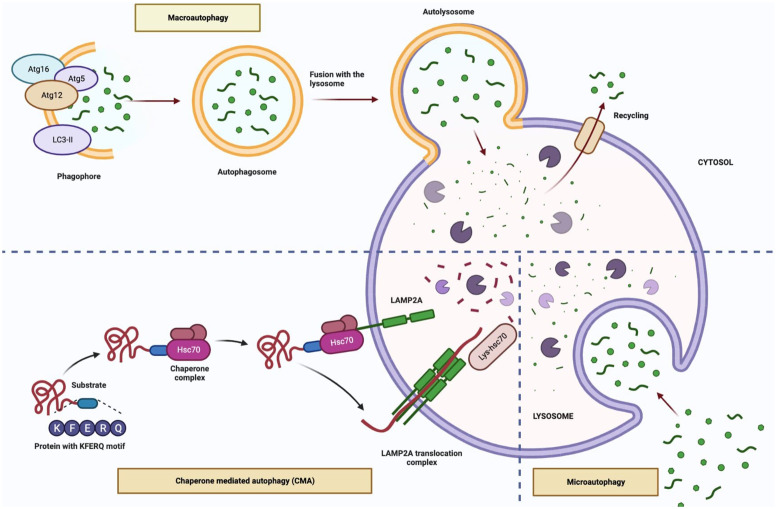
The types of autophagy (Figure created with Biorender.com).

Macroautophagy can be induced in the presence of various stress factors such as hypoxia, starvation, presence of damaged protein or organelles. This type of autophagy involves 5-steps including initiation, nucleation, elongation, fusion and degradation. In the initiation stage, inhibition of mTOR, the master regulator of autophagy, leads to activation of autophagy. The nucleation stage involving the ULK1 complex (ULK1/2, ATG13 and FIP200) and the class III PI3K complex (Beclin 1, ATG14L, p150) participates in phagophore formation. The phagophore membrane is closed via the ATG12 conjugation system (ATG12, ATG5 and ATG16L) and the LC3-II conjugation system in the elongation stage. This structure, called the autophagosome, contains the autophagic cargo. Finally, in the degradation stage, the autophagosome fuses with the lysosomal membrane and the autophagic cargo is sequestered by lysosomal hydrolases (Refs 15, 16, 17). In macroautophagy and microautophagy (also called endosomal microautophagy in mammals), cargo transport to the lysosome is vesicle-mediated, whereas in CMA it occurs through specific receptors (Refs 18, 19). In CASA, which is one of the selective macroautophagy types, polyubiquitinated aggregate proteins are transmitted to the autophagosome by helper chaperone Bcl2-related athanogen 3 (Bag3) and cytosolic chaperone heat shock cognate 70 kDa protein (HSC70) without binding to a pentapeptide amino acid motif (KFERQ)-like motif (Ref. 18). CMA is a variant of autophagy defined only for proteins carrying a specific targeting motif in the KFERQ amino acid sequence. The KFERQ-like motif is recognised by the cytosolic chaperone HSC70 and translocates to the substrate lysosome by the lysosome-associated membrane protein type A (LAMP2A) receptor. CASA is distinct from CMA, by being ubiquitin-independent (Fig. 1) (Refs 20, 21).

The processes of autophagy are related to aging in terms of biochemical regulation of cells. It has been reported that the decrease in gene expression of ATG5, ATG7, and Beclin-1 with age in humans is a result of decreased stimulation of macroautophagy (Ref. 22). Activation of macroautophagy as a result of disruption in the Beclin-1-BCL2 complex prolongs the lifespan of mice (Ref. 23). While the impairment of macroautophagy in cells such as neurons, melanocytes, and fibroblasts increases aging (Ref. 24), chemical regulation of macroautophagy in tendon stem cells decreases senescence (Ref. 25). Although the mechanism of microautophagy is well elucidated, the information on the relationship between microautophagy and aging is quite insufficient. One of the limited studies has shown that the accumulation of protein and lipid peroxidation products via microautophagy increases with aging (Ref. 26). The accumulation and aggregation of proteins known as CMA substrates are associated with the pathogenesis of aging and its accompanying diseases. Studies have shown that an increase in LAMP2A levels improves the reduction in CMA associated with aging (Ref. 27). Moreover, in previous preclinical studies, impaired CASA was shown to cause Z disc disintegration and progressive muscle weakness in flies, mice, and human, revealing the importance of chaperone-assisted degradation for the preservation of cellular structures of muscle tissues, thereby shedding light on aging (Ref. 21).

## Hallmarks of aging

Hallmarks of aging are mainly loss of proteostasis, mitochondrial dysfunction, disordered nutrient perception, genomic instability, telomere attrition, cellular senescence, cellular stemness.

### The role of autophagy in the loss of proteostasis

During the aging process, a decrease in the proteostasis activity of many cells and tissues is observed. Loss of proteostasis, a key feature of aging, is characterised by the appearance of misfolded or aggregated proteins. On the other hand, autophagy is capable of detecting proteotoxic stress and developing an appropriate response. Autophagy, which plays an important role in removing misfolded or unfolded protein aggregates, is impaired with age. With this degradation, protein aggregates accumulate and cause proteotoxicity (Ref. 28).

### Autophagy in mitochondrial dysfunction

The reduced mitophagy (known as clearing damaged mitochondria) associated with aging in mitochondria also leads to decreased energy production. Autophagy plays a key role in cleaning damaged mitochondria. Dysregulation in the autophagy mechanism causes mitochondria accumulation and thus oxidative stress (Ref. 29).

### Autophagy and cellular stemness

Autophagy plays a major role in the ability of adult stem cells to self-renew and differentiate into specialised cells. The decrease in autophagic activity with age results in a decrease in stem cells (Ref. 30).

### Autophagy and senescence

Oxidative stress, DNA damage, telomere shortening lead to cellular senescence. While autophagy over-stimulation induces cell death, its deficiency can trigger cellular senescence (Ref. 31).

### Autophagy and genomic instability

UV light, toxic chemicals, agents such as reactive oxygen species (ROS), mutations in nuclear and mitochondrial genes cause DNA damage. Weakening of the DNA repair mechanism and accumulating DNA damage trigger cellular senescence (Ref. 32).

### Autophagy and telomere attrition

Damage to telomeres or a decrease in their activity is directly related to the aging process (Ref. 33). An increase in cytoplasmic vacuoles and autophagy-related ATG5, ATG12 and LC3 proteins was observed in telomere-defected cells (Ref. 34).

## Molecular mechanisms of autophagy

Many signalling pathways have been identified to be responsible for regulating longevity and aging. Studies have shown that PI3K/AKT/mTOR, AMP-activated protein kinase (AMPK), MAPK, SIRT1, FoXO, signalling pathways are important in the regulation of autophagy as well as cell aging and metabolic disorders.

The PI3K/AKT/mTOR *axis* induced by growth factors is an essential signalling pathway in cancer cell growth in nutrient-rich conditions. In nutrient deficient conditions, inhibition of this pathway promotes autophagy (Ref. 35). TOR is an evolutionarily conserved serine/threonine kinase mainly localised in cytoplasm that regulates various cellular functions, including cell metabolism, survival, cytoskeleton, and autophagy (Ref. 36). mTOR is encoded by a single gene and there are two types of mTOR complexes as the protein product, functionally and structurally: mTOR complex 1 (mTORC1) sensitive to rapamycin and mTOR complex 2 (mTORC2) insensitive to rapamycin (Ref. 37). mTORC1 consists of mTOR, raptor (regulatory-associated protein of mTOR), mLST8 (mammalian lethal with sec-13 protein 8), PRAS40 (proline-rich AKT substrate 40 kDa), Deptor (the DEP domain containing mTOR-interacting protein), and TTI1/TEL2 (Ref. 38). mTORC1 is responsible for negative regulation of cell growth, differentiation and autophagy. Phosphorylation of mTORC1 inactivates ULK/ATG1, which promotes autophagy, increases anabolism, and promotes cell growth. Rapamycin is a well-known inhibitor of mTOR and also an inducer of macroautophagy. The mTORC2 consists of mTOR, mLST8, rictor, mSIN1, protor, and Deptor subunits. mTORC2 is mainly involved in the insulin signalling pathway and indirectly regulates autophagy (Ref. 39). The activity of mTORC1 is regulated by the PI3K/AKT/mTOR signalling pathway as well as by the AMPK and Ras-dependent MAPK pathways (Ref. 40). It was shown that regulation of autophagy dependent on the AMPK-mTOR signalling pathway can delay cardiomyocyte senescence (Ref. 41). It was also determined that overactivation of mTOR leads to impaired autophagy and results in premature aging (Ref. 42). ULK1, one of the important autophagy proteins, is a serine/threonine kinase and cellular autophagy is triggered by ULK1 binding to ATG13, FIP200 and ATG101 proteins to form a complex (Ref. 43). Under normal energy conditions, mTORC1 phosphorylates ULK1 and AMPK cannot activate ULK1. Cell autophagy is inhibited because it cannot form complexes with inactive ULK1, ATG and FIP200. In the absence of nutrients in cells, AMPK is activated, mTOR is inhibited, and autophagy is activated (Ref. 44). Consequently, the activated mTOR pathway inhibits the activation of ULK1 to induce senescence by inhibiting autophagy. When AMPK, the negative regulator of mTOR, is activated, it either activates ULK1 or inhibits mTOR and can delay aging by initiating autophagy.

**Figure 2.**
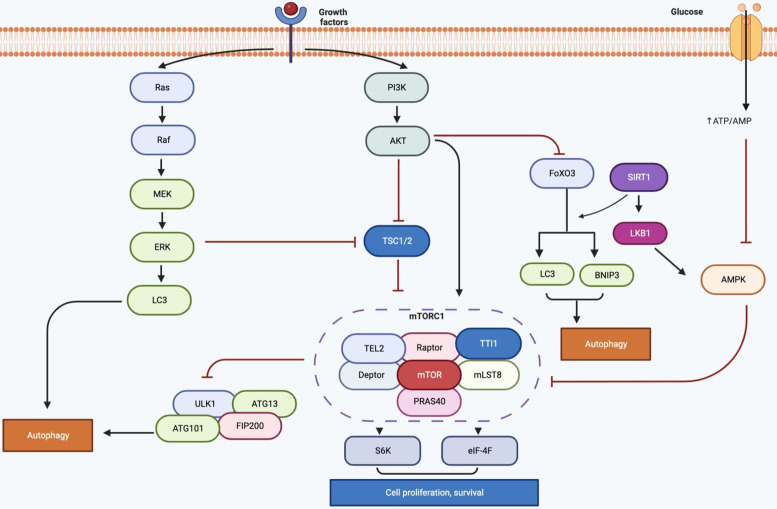
The molecular signalling mechanisms of autophagy (Figure created with Biorender. com).

SIRT1 is the mammalian homologue of Sir2 (silent information regulator) protein, known as stress resistance and longevity factor in yeasts. SIRT1 is the most studied sirtuin of seven human sirtuins (Ref. 45). SIRT1 is a class III protein deacetylase and uses nicotinamide adenine dinucleotide (NAD) as a cofactor to deacetylase lysine residues, thereby silencing genes, which can delay aging, reduce inflammation, improve energy metabolism. It was revealed that SIRT1 can form complexes with autophagy-related ATG5, ATG7, LC3 proteins and regulate autophagy by causing their direct deacetylation (Ref. 46). Calorie restriction delays the onset of age-related diseases such as NDs, cardiovascular diseases, and diabetes, therefore it is the only strategy to extend lifespan in many organisms (Ref. 47). Although the mechanism of extension of lifespan is unclear, it has also been determined that autophagy is increased during calorie restriction and energy deficiency (Ref. 48). SIRT1 level in humans decreases with age, therefore it causes cellular proteins misfolding and cell damage. SIRT1 and AMPK are positive regulators of each other. NAD expression is increased by AMPK and activates SIRT1 (Ref. 49). Calorie restriction also induces activation of SIRT1 with an anti-aging effect. The FoXO family of transcription factors (FoXO1, FoXO3a, FoXO4 and FoXO6) is also an important regulator of cellular metabolism and proliferation (Ref. 50). Recent studies have shown that signalling pathways related to SIRT1 and FoXO3 have an important role in the regulation of autophagy. SIRT1-mediated activation of FoXO1 induces expression of autophagosomes, and Rab7 (GTPase) which is highly important in autophagosome formation (Ref. 51). Overexpression of Rab7 also triggers autophagy, and activated FoXO3 protein increases the expression of autophagy-related proteins (LC3 and BNIP3) (Fig. 2) (Ref. 52).

## Autophagy and age-related diseases

Cellular components that have been implicated in the aging process have been linked to progressively impaired autophagy. In the elderly, dysfunctional autophagy may play a role in age-related disorders such as cancer, cardiovascular and neurological diseases and metabolic syndrome. Thus, restoring defective autophagy to its healthy state may aid in preventing age-related diseases and boosting longevity (Ref. 34).

### Autophagy in cancer

Cancer occurs when the balance between cell proliferation and death is lost, and more cells multiply than die or differentiate (Ref. 53). Unlike apoptosis, autophagy helps maintain homoeostasis by reversing intracellular molecules in the absence of nutrients or in cellular stress (Ref. 54). Basal autophagy works as a tumour suppressor mechanism during tumorigenesis; excessive autophagy works as a survival pathway in certain cancers (Ref. 55). Unlike normal cells, basal levels of autophagy are high in tumour cells, stimulated in hypoxic regions, and provide a constitutive survival advantage (Ref. 56). Therefore, inhibition of autophagy may prevent the survival of tumour cells and may be a new target for cancer therapy (Ref. 57) (Table 1).

**Table 1.** Studies on autophagy in cancer

Disease	Model	Results	References
Breast cancer	Beclin-1 gene transfer in autophagy-defective yeast and human MCF7 breast carcinoma cells with targeted degradation of Agp6/vps30	Beclin-1 is a functional homologue of Apg6/Vps30 and by using gene-transfer techniques promotes autophagy in autophagy-defective yeast, and in human MCF7 breast carcinoma cells.	(Ref. 59)
Brain cancer	mRNA expression of autophagy gene Beclin-1 in human brain tumours	Low expression has been observed in most high-grade glial tumours and atypical meningiomas, and Beclin-1 is important in the pathobiology of brain tumours.	(Ref. 61)
Skin cancer	UVRAG-mediated UV protection tested *in vivo* by comparing tissue loss in the irradiated eye with tissue loss in the untreated eye in *Drosophila pupal* retina model.	UVRAG is a regulator of CRL4DDB2-mediated nucleotide excision repair (NER) which is the primary repair pathway of UV-induced DNA damage, an important risk factor in skin cancer.	(Ref. 62)
Ovarian cancer	ATP6V0A2 wild-type and ATP6V0A2 V823 mutant cell lines were tested with PHY34 which is induced apoptosis in ovarian cancer cells by late-stage autophagy inhibition.	PHY34 is a promising molecule that could be used in cancer therapy targeting the ATP6V0A2 subunit to induce autophagy.	(Ref. 63)
Ovarian cancer	Expression of ubiquitin-conjugating enzyme E2T (UBE2T) and its association with clinicopathological features and prognosis in ovarian cancer patients were evaluated.	Knockdown of UBE2T significantly inhibits cell growth and proliferation by autophagy.	(Ref. 64)
Colon, pancreas prostate and melanoma cancers	Mouse colon carcinoma CT26 cells, mouse pancreatic carcinoma (pan02), melanoma (B16F10), and prostate cancer (RM-1) treated with shikonin, HCQ, doxorubicin hydrochloride (DOX), oxaliplatin	Combining autophagy inhibitor and ICD inhibitor could be a potential cancer treatment method.	(Ref. 65)
Gallbladder carcinoma	SGC-996 and GBC-SD cell lines induced by 5-FU.	5-FU induces protective autophagy and creates chemoresistance.	(Ref. 73)
Fibrosarcoma	HT1080 was treated with spautin-1 after silencing of the USP10 and USP13 genes in human fibrosarcoma cells.	Spautin-1 inhibits the unfolded protein response under 2-deoxy-D-glucose and glucose starvation stressed conditions.	(Ref. 75)
Hypopharyngeal squamous cell carcinoma (head and neck cancer)	BALB/c nude mice with a subcutaneous injection of human hypopharyngeal FaDu cells	CQ-inhibited autophagy by increasing the levels of LC3 and p62 and enhanced the efficacy of cisplatin	(Ref. 76)

Autophagy is used to provide components and energy to cells for basal survival in each cell (Ref. 54). Autophagy is controlled by class I PI3K/mTOR and AMPK signalling pathways. During nutrient availability (growth factors and amino acids), mTOR kinase, a target of rapamycin, suppresses autophagy. Mutations in the PI3K/AKT/mTOR pathway, in which autophagy is negatively regulated, may lead to oncogenic transformations by increasing signalling in the pathway (Ref. 58).

Beclin-1, which has a central role in autophagy, can function as a tumour suppressor (Ref. 59). Beclin-1 is monoallelic deleted in many cancers, such as breast, ovarian, brain, and prostate cancers, and low protein levels have been found in the tumours of these cancers. Studies in mice have shown that heterozygous disruption of beclin-1 increases permeability to spontaneous tumour development (such as lymphomas, lung carcinomas, hepatocellular carcinomas, and mammary precancerous lesions), whereas Beclin-1 restoration in MCF-7 cells reduces proliferation and tumorigenesis (Refs 59, 60, 61). Bif-1 plays a role in inducing autophagosome formation in autophagy and interacts with Beclin-1 via UVRAG. UVRAG proteins have been observed to be abnormal or deleted in various types of cancer, such as colorectal and gastric cancer. It is also a tumour suppressor candidate because it reduces proliferation and tumour size in cases of excessive UVRAG expression (Ref. 62). It has been reported that the MAP1-LC3 gene, which encodes LC3-II located in the inner and outer membrane of the autophagosome and functions in autophagy substrate selection and autophagosome biogenesis, is frequently deleted in the liver, breast, prostate, and ovarian cancers (Ref. 63). Thus, it can be considered that autophagy is both a tumour suppressor and promoter (via protective autophagy) mechanism, tumours with autophagy deficiency grow faster and this situation increases exponentially with defects in apoptosis.

Modulation of autophagy in cancer therapy is a promising potential strategy. Autophagy is also a protective mechanism in cancer cells during anticancer treatment. The common treatment strategy in cancer treatment is chemotherapy, but the development of chemoresistance (such as the protective autophagy mechanism) limits the success rate. Some autophagy regulators, such as the best-known mTOR inhibitors rapamycin and its derivatives (temsirolimus and everolimus), chloroquine (CQ), and hydroxychloroquine (HCQ), are also used in cancer treatment.

Rapamycin, an mTOR inhibitor, was developed to inhibit proliferation signals in the PI3K/AKT/mTOR pathway, which is important for cell growth and proliferation in multiple tumour types (Ref. 64). Moreover, rapamycin may sensitise cancer cells to radiation therapy and chemotherapeutic agents such as adriamycin, cisplatin and hormonal therapies (Ref. 65). In addition rapamycin is used in rare lung diseases, coronary stents (Ref. 66). Temsirolimus was the first mTOR inhibitor class drug to be shown to improve survival in patients with advanced renal cell carcinoma (Refs 67, 68). Increased PI3K/AKT expression and activation are common in renal cell cancer and this drug is used in the first-line treatment of metastatic renal cell cancer with poor prognosis (Ref. 66). It is also used in the treatment of relapsed or refractory mantle cell lymphoma in the European Union. Everolimus is used in combination with exemestane in the treatment of advanced neuroendocrine tumours and breast cancer (Refs 69, 70). In preclinical studies in bladder cancer and pancreatic adenocarcinoma, it has been shown that CQ and HCQ can suppress cancer cell growth by inhibiting autophagy. Several studies have demonstrated that these reagents promote apoptosis through suppression of autophagy and enhance the therapeutic effects of chemotherapy by inhibiting autophagy-mediated resistance seen in therapy (Refs 70, 71). Lys05, a water-soluble analogue of HCQ, showed higher anticancer effects than HCQ in low doses of melanoma and colon cancer xenograft models (Ref. 72). 5-Fluorouracil (5-FU), which is used in solid cancers such as colorectal, breast, and pancreatic cancer, has limited efficacy in the treatment because it induces protective autophagy and creates chemoresistance (Refs 73, 74). Spautin-1, SAR405 are newly developed autophagy-related anticancer drugs (Ref. 75).

### Autophagy in cardiovascular diseases

Autophagic activity is implicated in the regulation of mammalian heart development in the early stage, maintenance of cardiac structure and function under normal conditions, in the onset and progression of cardiovascular diseases including ischaemia/reperfusion (I/R) and heart failure (Ref. 4). In addition, autophagy is important in delaying cardiac aging (Ref. 77).

Activation of autophagy during myocardial ischaemia ensures the maintenance of energy substrates and removal of damaged mitochondria that may initiate apoptosis by causing oxidative stress (Ref. 78). Autophagy activation during ischaemia relies on AMPK mediated inhibition of the mTORC1 pathway (Ref. 79). Inhibition of autophagy during chronic ischaemia can cause cardiomyocyte death by activating apoptosis. Therefore, induction of autophagy during myocardial ischaemia may be protective.

During reperfusion, tissues exposed to ischaemia are reloaded with nutrients and oxygen. Reperfusion injury is defined as damage to tissues or organs during the re-bleeding period following the ischaemic period. Studies have shown that autophagy is a key regulator with a dual role for I/R and is activated during I/R (Ref. 80). AMPK activation is involved in autophagy resulting from ischaemia, whereas activation of Beclin 1 is involved in autophagy during reperfusion. Autophagy plays different roles during ischaemia and reperfusion; while protective during ischaemia, it could be harmful during reperfusion (Ref. 79). (Table 2).

**Table 2.** Studies on autophagy in cardiovascular diseases

Disease	Model	Results	References
Hypoxia	Hypoxic mouse embryo fibroblasts (MEFs) cells	Hypoxia-induced BNIP3 triggers autophagy by increasing free Beclin-1 levels	(Ref. 78)
Ischaemia and reperfusion	Transgenic mice and cardiac myocytes incubated in glucose-free medium were used.	Autophagy activation during ischaemia relies on AMPK mediated inhibition of the mTORC1 pathway. Autophagy plays dual roles during ischaemia and reperfusion: while protective during ischaemia, it could be harmful during reperfusion.	(Ref. 79)
Cardiomyopathy	cardiac-specific ATG5-deficient mice.	Deficiency of ATG5 causes loss of cardiac-specific autophagy and cardiomyopathy in mice.	(Ref. 81)
Ischaemia and reperfusion	H9C2 cells for hypoxia/reoxygenation and adult male C57/B6 mice for ischaemia/reperfusion	ischaemic postconditioning helped autophagy against I/R injury by the activation of nNOS/AMPK/mTOR pathway.	(Ref. 80)

The heart's first response to various stresses, such as hypertension, aortic stenosis, is hypertrophy, and if this situation continues, heart failure may develop (Ref. 81). Furthermore, age-related progressive loss of autophagic activity leads to the development of hypertrophy (Ref. 77). It remains unclear whether the altered autophagy observed in cardiac hypertrophy and heart failure is beneficial or harmful. *β*-adrenergic stimulation, which induces cardiac hypertrophy and heart failure, inhibits autophagy and stimulates apoptosis (Ref. 80). The deficiency of ATG5 causes cardiac hypertrophy by inhibition of autophagy with increased ubiquitination levels, while overexpression of ATG5 increases autophagy levels with Beclin-1 function and extends lifespan (Ref. 82). In conclusion, basal autophagy in the heart under normal conditions is a homoeostatic mechanism for maintaining cardiac structure and function.

The prevalence of autophagic activation in cardiac diseases has led to studies in this area for therapeutic targets. In the conjectural applications of cardiovascular diseases, 8-methylchroman-7-ol derivatives and glycosylated anti-tumour ether lipids inhibit autophagy; Rubicon, which binds the class III PI3K/Vps34–Beclin 1 complex, and AP23573, a phosphorus-rapamycin analogue, are used in the regulation of autophagy (Ref. 83).

### Autophagy in neurological aging

Autophagy is important for the maintenance of neuronal homoeostasis by mediating the clearance of unfolded or misfolded protein aggregates.

Age-related decrease in autophagy may lead to accumulation of intracellular toxic wastes (Ref. 28). This condition is characterised by aggregated and abnormally deposited proteins in NDs. Apart from protein aggregation, mitochondrial dysfunction and decreased lysosomal activity accompany dysregulated autophagy in NDs. Aging and neuronal autophagy dysregulation increase the incidence of NDs such as AD, PD, HD (Table 3). In particular, misfolded protein accumulation and disruption of the autophagy pathway due to mTOR are seen in these diseases. With aging, there may be decreases in the ubiquitin-proteasome system and autophagy-lysosomal pathway, which are associated with the clearance of misfolded proteins in the brain (Ref. 84). Measures to eliminate the disorders in the autophagy mechanism may contribute to the prevention of age-related diseases. Gene polymorphisms involved in autophagy mechanisms contribute to aging-related neurodegeneration. Plaques formed as a result of tau protein accumulation in AD, mutant *α*-synuclein proteins (Lewy bodies) in PD, and mutant Huntingtin protein (mHtt) accumulation in the cytoplasm are seen in HD. Autophagy plays an active role in the clearance of these proteins. There is evidence that overexpression of ATG5, which is involved in autophagy induction, activates autophagy and increases survival. However, it was shown that there was a decrease in the expression of the ATG7 and Beclin-1 genes. In this case, it can be concluded that the decrease in autophagy activation decreases with aging (Ref. 22).

**Table 3.** Studies on autophagy in neurodegenerative diseases

Disease	Model	Results	References
AD	APP transgenic mice Becn1^+/–^ transgenic mice	Decreased Beclin-1 expression; Increased intraneuronal A*β* accumulation	(Ref. 96)
AD	Triple transgenic AD (3xTg-AD) mice	Parkin-mediated reduction of A*β* levels	(Ref. 97)
AD	Becn1^F121A/F121A^ knock-in mice	Hyperactivation of autophagy by inhibition of Beclin-1-BCL2 binding	(Ref. 98)
AD	Senescence accelerated mouse prone 8 (SAMP8) mice	Increased expression levels of LC3-II in hippocampus and cortex; Decreased expression levels of Beclin-1 expression	(Ref. 99)
AD	TSC2 heterozygous mice	Regulation of tau phosphorylation and degradation by mTOR; Decreased expression levels of LC3-II in hippocampus; Decreased levels of ATG7 and ATG5/ATG12	(Ref. 100)
AD	Becn1^+/–^ transgenic mice APP express transgenic mice	Decreased neuronal autophagy and disruption of lysosomes intraneuronal A*β* accumulation	(Ref. 96)
AD	Triple transgenic AD (3xTg-AD) mice transfected with mutant APP	A*β* accumulation, hyperactivation of mTOR	(Ref. 101)
PD	LRRK2 G2019S knockout mice; LLRK2 knockout mice; LRRK2 D1994S knockout mice	Increased LC3-I, p62, LAMP2 and GAPDH levels Decreased p-mTOR levels; Downregulation of mTOR and TFEB expression; LAMP2 accumulation	(Ref. 102)
PD	1-methyl-4-phenyl-1,2,3,6-tetrahydropyridine (MPTP) mouse model	Loss of dopaminergic neurons and improved motor functions	(Ref. 103)
PD	Rotenone-induced neurotoxicity in SH-SY5Y cells	Increased mTOR and Beclin-1	(Ref. 104)
PD	A transgenic mouse model (TgM83) expressing the human A53T *α*-synuclein mutation	Neuroinflammation, metabolic dysregulation, and photoreceptor cell death by accumulation of *α*-synuclein	(Ref. 105)
PD	A53T *α*-synuclein transgenic mice	Decreased the cell damage induced by overexpressing A53T *α*-synuclein Activation of JNK/Bcl-2-mediated autophagy pathway	(Ref. 106)
HD	N171-82Q and R6/1 mouse overexpressing wild-type *α*-synuclein and carrying a deletion in the Snca locus	Impaired autophagy by *α*-synuclein overexpression Accumulation of p62	(Ref. 107)
HD	*α*-syn knockout R6/1 HD mice	Body weight loss and early motor phenotype	(Ref. 108)
HD	C6R mHtt mice model	Degradable mHtt impairs basal autophagy	(Ref. 109)
HD	Alfy knockout mice and BACHD mice	Increased age of onset and progression of HD	(Ref. 110)
HD	HD knock-in Mouse model (HdhQ^7,111^)	Modulation of autophagy and Htt protein levels	(Ref. 111)
HD	R6/2 transgenic mice	Increased mHtt phosphorylation by TBK1 expression	(Ref. 112)
HD	R6/2 transgenic mice	Improved fine motor control treatment with mitoQ	(Ref. 113)
HD	Activation of AMPK by A769662 in STHdh cells and mouse embryonic fibroblasts	Increased expression of LC3 and p62	(Ref. 95)
HD	Q175 HD mice MEF, HEK293T, HCT116, and HeLa cells	Enhanced autophagy by ULK1-mediated phosphorylation of Atg14	(Ref. 114)

AD, Alzheimer's Disease; PD, Parkinson Disease; HD, Huntington's Disease.

#### Alzheimer's disease

AD is a ND characterised by the accumulation of extracellular *β*-amyloid (A*β*) plaques and intracellular neurofibrillary tangles containing hyperphosphorylated tau (Ref. 5). Only 5% of AD is familial and results from mutations in presenilin 1, presenilin 2, and amyloid precursor protein (APP). A*β* and tau protein are substrates for autophagy. An abundance of autophagic structures containing these substrates is observed in AD. In addition, it has been shown that abnormal conformations of A*β* reduce lysosomal amplification, lead to synaptic defects and increase the course of the disease (Ref. 85). Neurofibrillary tangles caused by mutant tau proteins can block CMA, resulting in decreased autophagy. Beclin-1, an autophagy-related protein, decreases with age in the brains of AD models, and increased levels of Beclin-1 ameliorate amyloid pathology.

Due to the morphological structure of nerve cells, the levels of clearance by autophagosomes may be different compared to other cells. The importance of autophagy in these cells is due to their post-mitotic nature. Autophagy is very important in cell survival as it can remove toxic aggregates in post-mitotic nerve cells. It has been found that autophagosome clearance is impaired in late-stage disease in neuron cells from patients with AD only. In some studies, it has been reported that the levels of some autophagy genes are increased in AD brains (Ref. 22).

#### Parkinson disease

PD is a ND caused by selective loss of dopaminergic neurons and decreased dopamine content in the striatum (Ref. 86). This loss can be induced by mitochondrial toxins such as 1-methyl-4-phenyl-1,2,3,6 tetrahydropyridine (MPTP) and the complex I inhibitor rotenone. As in AD, mutations that cause PD can directly cause disruption of autophagy. Some of these mutations are *α*-synuclein (SNCA or PARK1), also known as Lewy bodies, Parkin (PRKN or PARK2), ubiquitin carboxy-terminal hydrolase L1 (UCH-L1), PTEN-induced putative kinase 1 (PINK1), protein deglycase DJ-1 (PARK7) and leucine-rich repeat kinase 2 (LRRK2) (Ref. 87). Furthermore, many PD-related gene mutations can cause loss of function in the autophagy-lysosome pathway (ALP). Because these genes play a role in mitophagy, mutations in these genes cause insufficient mitochondrial quality control (Ref. 88). Two major mutations in *α*-synuclein (A53T and A30P) are associated with PD (Ref. 89). Mutant *α*-synuclein causes disruption of CMA activity, inhibition of autophagosome formation and disruption of lysosomal degradation (Ref. 90). Increased expression of mTOR, one of the important autophagy pathways, has been reported after *α*-synuclein accumulation in patients with PD. Apart from aging and genetic mutations, PD may also develop due to dopaminergic neuron-specific toxins such as 6-hydroxydopamine (6-OHDA), 1-methyl-4-phenyl-1,2,3,6 tetrahydropyridine (MPTP) and rotenone.

#### Huntington's disease

HD is an autosomal dominant ND caused by long CAG trinucleotide expansion (over 37 repeats) in the Htt gene (Ref. 91). In patients with HD, an abnormally long polyglutamine-encoding CAG trinucleotide expansion produces perinuclear cytoplasmic aggregates and intranuclear inclusions. This results in mutant Htt, an autophagic substrate, ingestion of defective autophagic substrate into autophagosomes. In addition, this expansion of polyglutamine causes misfolded proteins or accumulation of protein aggregates. Deletion of polyglutamine in the Htt gene has been shown to improve disease symptoms and increase autophagosome formation (Ref. 92). In addition, the decrease in Beclin-1 expression with aging may lead to the accumulation of mutant Htt (Ref. 93). It has also been reported that the mTOR pathway plays a role in HD pathology. In a recent study, they identified the Homeodomain Interacting Protein Kinase 3 (HIPK3) gene as a negative modulator of autophagy and a positive regulator of mHtt expression levels in HD cells. It is considered that modulation of mHtt by HIPK3 can be a therapeutic target for HD (Ref. 94). Stimulation of autophagy usually occurs by inhibition of mTOR. However, since mTOR is involved in many cellular processes, its inhibition can lead to unexpected effects. Walter *et al*. showed that AMPK activation causes increased expression of LC3-II and p62, and induced autophagy in an mTOR-independent manner. This resulted in decreased aggregates containing mHtt and cell viability (Ref. 95).

### Autophagy in obesity and diabetes

Obesity, which is associated with excessive calorie intake, is a chronic disease that occurs as a result of many genetic and environmental factors, affecting the metabolism and physiology of organs (Ref. 115). Since triglycerides are stored in adipose tissue and metabolised by the liver, adipose tissue is at the centre of obesity and metabolic diseases (Ref. 116). Autophagy, on the other hand, is a physiological process that removes damaged organelles, misfolded proteins, and lipids in case of excessive calorie intake. Autophagy is a highly sensitive mechanism to excessive calorie intake. Suppression or increase of autophagy is seen in metabolic diseases such as obesity and diabetes (Ref. 6).

Insulin resistance, which develops due to the increase in calorie intake, suppresses the activity of the mTOR pathway; this initiates the autophagy process in adipose tissues. However, it has been determined that calorie restriction increases autophagy activity and, accordingly, insulin sensitivity in obese individuals. In addition, an increase in the LC3 level and the number of autophagosomes in the fat cells of obese patients was observed. In another study, it was determined that the mRNA expression level of the autophagy gene ATG5 was higher in patients with higher BMI and larger visceral adipose tissue (Ref. 117). Obesity was shown to stimulate inflammation by causing hypothalamic resistance to insulin and leptin hormones through an inhibitor of the Nuclear Factor kappa B (NF-kB) pathway and induces hypothalamic dysfunction (Ref. 118). Therefore, hypothalamic dysfunction can be considered to play a role in the pathophysiology of obesity and diabetes. In studies, hypothalamic inhibition of autophagy via siRNA-mediated ATG7 suppression was achieved in mice fed a high-fat diet, resulting in increased energy consumption. In other words, feeding with HFD in mice caused disruption of lipolysis and insulin resistance. It has also been observed that hypothalamic autophagy is impaired in HFD-induced obesity (Ref. 119).

Diabetes is a metabolic disease identified as chronic hyperglycaemia. There are two main clinical types, insulin-dependent (Type 1) and insulin-independent (Type 2) (Ref. 120). Type 1 diabetes mellitus (T1DM) is a disease that develops by autoimmune destruction of pancreatic beta cells. It is known that insulin reduces tau phosphorylation and autophagy by inhibiting the PI3K/AKT signalling pathway-mediated Glycogen synthase kinase-3 (GSK-3). Phosphorylation of tau protein is increased in T1DM with insulin deficiency (Ref. 121). On the other hand, insulin resistance, hyperglycaemia and relative insulin deficiency determine the development of Type 2 diabetes mellitus (T2DM). The increase of adipose tissue in T2DM causes insulin resistance by activating the mTOR pathway. mTORC1 is activated by high amounts of glucose, fatty acids and amino acids. Moreover, insulin is a hormone that inhibits autophagy (Ref. 122). Insulin resistance causes autophagy activation in adipose tissues of obese patients. In the previous studies, it was demonstrated that insulin resistance was induced and autophagy was inhibited by decreasing the expression levels of autophagy-related LC3, ULK2, ATG12 proteins in livers of mice fed a high-fat diet (Ref. 123). Studies on autophagy in obesity and diabetes are summarised in Table 4.

**Table 4.** Studies on autophagy in obesity and diabetes

Disease	Model	Results	References
T2DM	*In vivo* early stage of type 2 diabetic nephropathy mice model	Regulating autophagy and apoptosis in mice through the suppression of microRNA-383-5p	(Ref. 124)
T1DM	*In vivo* Streptozotocin (STZ)-induced type 1 diabetes rats model	Increased in tau protein phosphorylation and DRP1 protein phosphorylated in the brain cortex	(Ref. 121)
T2DM	*In vivo* cPKC*γ* knockout diabetes mice model	Reduced LC3-II mRNA and protein level; increased p62 protein level	(Ref. 125)
T2DM	*In vivo* ATG7 knockout mice model specific for pancreatic *β* cells	Reduced glucose-stimulated insulin secretion and impaired glucose-induced cytosolic Ca2 + transients	(Ref. 126)
T2DM	*In vivo* genetic ablation of ATG7 specific for pancreatic beta cells in mice model	Resulted in degeneration of pancreatic islets and impaired glucose tolerance with reduced insulin secretion	(Ref. 127)
T2DM	*In vivo β* cell-specific ATG7 deficient mice model	Accumulation of polyubiquitinated protein aggregates associated with p62, formation of large insulin-containing inclusion bodies and decreased insulin secretion	(Ref. 128)
T2DM	*In vivo* insulin secretion-deficient Rab3A^−/−^ null mice model	Increased insulin degradation via macroautophagy	(Ref. 129)
T2DM	*In vivo β* cell-specific ATG7-deficient mice model	Reduced the expression of unfolded protein response (UPR) and antioxidants genes associated with ER stress	(Ref. 130)
T2DM	*In vivo* Zucker diabetic fatty rats model	Hyperglycaemia and oxidative stress increased accumulation of polyubiquitinated protein aggregates degraded by autophagy	(Ref. 131)
T2DM	*In vitro* ATG7-knockdown INS-1 rat insulinoma cells and *in vivo β* cell-spesific ATG7-deficient mice model	Obesity, increased blood glucose levels	(Ref. 132)
Obesity	*Ex vivo* siRNA-mediated ATG7 suppression in obese human adipose cells, *In vivo* obese leptin deficient mice model	Increased LC3-II, IL-1*β*, IL-6, and IL-8 mRNA and protein level	(Ref. 133)
Obesity	*Ex vivo* obese human tissue biopsies	Increased LC3-II/LC3-I ratio, LC3 and ATG5 mRNA level; decreased p62 and mTOR protein levels	(Ref. 134)
Obesity	*In vivo* high fat diet (HFD)-induced fat-1 transgenic mice model	Increased LC3-II, ATG12-ATG5; reduced p62 expression protein level	(Ref. 135)
Obesity	*In vivo* HFD-induced nonalcoholic fatty liver disease rat model	Increased Beclin-1, LC3-I/II, ATG3, ATG5, JNK phosphorylation; decreased mTOR phosphorylation	(Ref. 136)
Obesity	*In vivo* OLETF-diabetic obese rat model	Decreased ATG5, ATG6, ATG7 mRNA level and LC3 protein level	(Ref. 137)
Obesity	*In vivo* HFD induced-obese mice model	Defective insulin signalling and increased ER stress, reduced autophagy activation (ATG7 level)	(Ref. 138)
Obesity	*In vivo* HFD induced-obese mice model	Decreased LC3-I, LC3-II protein level, JNK phosphorylation; increased mTOR phosphorylation	(Ref. 139)
Obesity	*In vivo* HFD induced-obese mice model	Decreased LC3-II, Beclin-1 protein level; decreased autophagosome; increased p62 protein level	(Ref. 140)
Obesity	*In vivo* HFD induced-obese mice model	Increased LC3B1 and Atg7 protein level	(Ref. 141)

## Natural active compounds in longevity intervention

Age is the greatest risk factor for all major age-related pathologies. In recent years, it has been reported that the molecular mechanisms underlying aging are associated with cancer, cardiovascular diseases and neurodegeneration. It can also affect the same pathway, autophagy, as these diseases. Therefore, autophagy is gaining importance in the discovery of therapeutic interventions that promote healthy aging and increase longevity (Ref. 142).

Natural products were widely used to treat different medical conditions before the development of modern pharmaceuticals. Today they still serve as an important pool for the identification of new drug precursors against various diseases including cancer and chronic diseases, which become widespread with increasing age. The structural complexity and diversity of natural products provide a valuable resource for future drug discovery.

In the following section, we summarise the autophagic mechanisms of natural compounds in cancer, cardiac diseases, neurological aging, diabetes and obesity.

### Autophagic mechanisms of natural compounds in cancer

**Figure 3.**
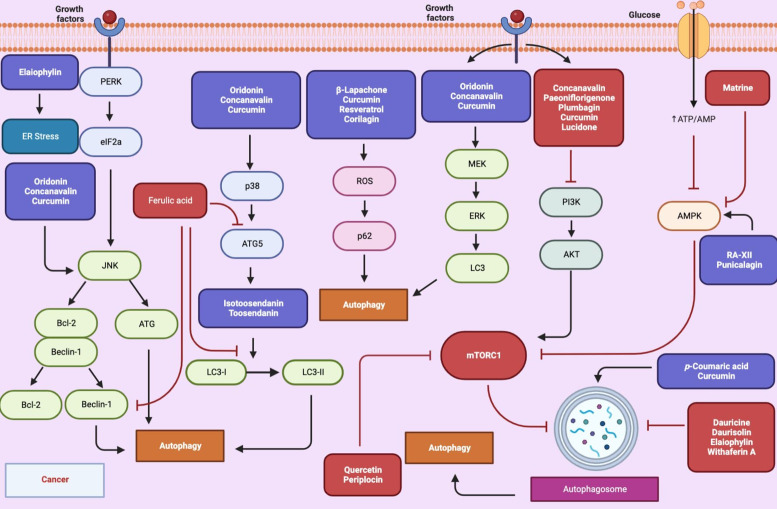
Autophagy-related pathways regulated by natural compounds in cancer (Figure created with Biorender.com).

Natural compounds have been identified as potent inducers or inhibitors of autophagy to exhibit antitumour mechanisms, opening up new therapeutic regimens against cancer. Some studies suggest that autophagy exerts a tumour-suppressing role, while others suggest a tumour-promoting role. This complexity of autophagy in cancer biology may arise from different contexts such as stress types, tumour staging and cancer types. *In vitro* studies on different groups of natural compounds modulating autophagy and molecular target mechanisms were summarised in Table 5 and Figure 3.

**Table 5.** *In vitro* studies on natural compounds modulating autophagy in various cancers signalling pathways

Group	Compound name	Cancer type and cell lines	Autophagic target	Molecular target	References
Alkaloids	Berbamine	Breast cancer (MCF7, MDA-MB-231)	Autophagy inhibition	Inhibits interaction of SNAP29 and VAMP8	(Ref. 143)
Alkaloids	Cepharanthine	Non-small cell lung cancer (NCI-H1975, NCI-H1650, HCC827, A549, NCI-H1299)	Autophagy inhibition	Inhibits maturation of cathepsin B and D	(Ref. 144)
Alkaloids	Dauricine and daurisolin	Cervical cancer (HeLa), Colon cancer (HCT 116), Non-small cell lung cancer (A549)	Autophagy inhibition	Inhibits autophagosome maturation	(Ref. 145)
Alkaloids	Liensinine	Breast cancer (MCF7, MDA-MB-231)	Autophagy inhibition	Dephosphorylation and mitochondrial translocation of DNM1L	(Ref. 146)
Alkaloids	Matrine	Liver cancer (HepG2, SMMC-7721)	Autophagy induction	p53/AMPK	(Ref. 147)
Cyclopeptides	RA-XII	Liver cancer (HepG2)	Autophagy inhibition	Inhibits AMPK/mTOR/p70S6K	(Ref. 148)
Diterpenoids	Oridonin	Cervical cancer (HeLa)	Autophagy induction	Suppress Ras and promote p38, MAPK, and JNK	(Ref. 149)
Flavonoids	Kaempferol	Gastric cancer (AGS, SNU-216, NCI-N87, SNU-638, MKN-74)	Autophagy as epigenetic regulator	Inhibits HDAC/G9a	(Ref. 152)
Flavonoids	Quercetin	Pancreatic cancer (MIA Paca-2, BxPC-3, AsPC-1, PANC-1)	Autophagy induction	Inhibits mTOR, proteasomal activity	(Ref. 153)
Indole derivatives	3,3′-Diindolylmethane	Gastric cancer (BGC-823, SGC-7901)	Autophagy as epigenetic regulator	Inhibits miR-30e leading to ATG5 activation	(Ref. 154)
Lectins	Concanavalin	Cervical cancer (HeLa)	Autophagy induction	Decreases PI3K/AKT/mTOR and increases MEK/ERK	(Ref. 150)
Lignans	Honokiol	Melanoma (B16/F-10, SKMEL-28)	Autophagy induction	Inhibits notch pathway resulting in autophagic cell death	(Ref. 155)
Monoterpenes	Paeoniflorigenone	Head and neck cancer (YD-10B)	Autophagy induction	Inactivates PI3K/AKT/mTOR/p70S6K	(Ref. 156)
Naphthoquinones	*β*-Lapachone	Glioblastoma (U87 MG, U343)	Autophagy induction	Induces ROS	(Ref. 157)
Naphthoquinones	Plumbagin	Non-small cell lung cancer (A549, H23)	Autophagy induction	Inhibits PI3K/AKT/mTOR	(Ref. 158)
Phenolic acids	Ferulic acid	Cervical cancer (HeLa, CaSki)	Autophagy induction	Decreases LC3-II, Beclin-1 and ATG12-ATG5	(Ref. 151)
Phenolic acids	*p*-Coumaric acid	Neuroblastoma (N2a)	Autophagy induction	Increases autophagosomes formation	(Ref. 159)
Polyketides	Elaiophylin	Multiple myeloma (Mutant TP53)	Autophagy inhibition	Increases ER stress	(Ref. 160)
Polyketides	Elaiophylin	Ovarian cancer (SKOV3, OVCAR3, A2780, CaOV-3, and SW626)	Autophagy inhibition	Inhibits autophagic flux	(Ref. 70)
Polyphenols	Curcumin	Glioblastoma (U87-MG, U373-MG)	Autophagy induction	Inhibits AKT/mTOR/p70S6K and activates ERK1/2	(Ref. 161)
Polyphenols	Curcumin	Prostate cancer (22rv1, LNCaP, (DU145, PC-3))	Autophagy induction	Modulates Wingless signalling pathway	(Ref. 162)
Polyphenols	Curcumin	Colon cancer (HCT116)	Autophagy induction	Activates of ERK1/2 and p38 MAPK	(Ref. 163)
Polyphenols	Curcumin	Oral cancer (OSCC)	Autophagy induction	Induces ROS production	(Ref. 164)
Polyphenols	Curcumin	Pancreatic cancer (PANC1, BxPC3)	Autophagy induction	Increases autophagosomes	(Ref. 165)
Polyphenols	Curcumin	Prostate cancer (PC3, DU145, LNCaP)	Autophagy as epigenetic regulator	Inhibits DNMT1 and DNMT3B	(Ref. 166)
Steroidal lactones	Withaferin A	Breast cancer (MCF7, MDA-MB-231, MDA-MB-468, T47D)	Autophagy inhibition	Inhibits lysosomal proteolytic activity	(Ref. 167)
Steroids	Periplocin	Pancreatic cancer (CFPAC1, PANC1)	Autophagy induction	Increases p-AMPK expression and decreases p-mTOR expression	(Ref. 168)
Stilbenes	Resveratrol	Breast cancer (MCF7, SUM159)	Autophagy induction	Decreases WNT/*β*-catenin	(Ref. 170)
Stilbenes	Resveratrol	Colon cancer (HT-29, COLO 201)	Autophagy induction	Induces ROS	(Ref. 171)
Stilbenes	Resveratrol	Ovarian cancer (NIH-OVCAR3)	Autophagy as epigenetic regulator	Activates ARH1 to inhibit IL-6 dependent cell migration	(Ref. 172)
Tannins	Corilagin	Gastric cancer (SGC7901, BGC823)	Autophagy induction	Induces ROS	(Ref. 173)
Tannins	Punicalagin	Glioblastoma (U87-MG)	Autophagy induction	Increases AMPK/p27	(Ref. 174)
Terpenoids	Lucidone	Pancreatic ductal adenocarcinoma (MIA Paca-2)	Autophagy inhibition	Inhibits the HMGB1/RAGE/PI3K/Akt	(Ref. 175)
Triterpenoids	Celastrol	Prostate cancer (NCaP, 22Rv1, DU145, PC-3)	Autophagy as epigenetic regulator	Inhibits AR/miR-101	(Ref. 176)
Triterpenoids	Isotoosendanin	Breast cancer (MDA-MB-231, BT549, 4T1)	Autophagy induction	Increases LC3B II/LC3B I ratio	(Ref. 177)
Triterpenoids	Toosendanin	Breast cancer (MDA-MB-231, BT549, 4T1)	Autophagy induction	Increases LC3B II/LC3B I ratio	(Ref. 177)

Alkaloids are a group of secondary metabolites that are extensively found in nature and have strong pharmacological activities. They can target the autophagy process in different cancer types including breast, lung, cervical, colon, liver and pancreatic cancer. Among the alkaloids, berbamine, cepharanthine, dauricine, daurisolin, liensinine inhibited the autophagy via different molecular target mechanism while matrine induced autophagy by p53/AMPK pathway (Refs 143, 144, 145, 146, 147). Natural cyclopeptide RA-XII suppresses protective autophagy through AMPK/mTOR/P70S6 K pathways in HepG2 cells (Ref. 148). Oridanin, concanavalin and ferulic acid induced autophagy and displayed an anticancer effect against cervical cancer (Refs 149, 150, 151). Kaempferol and quercetin are natural flavonoids that are found in many fruits, vegetables, and medicinal plants. They mediated autophagy in gastric and pancreatic cancer cell lines via inhibition of HDAC/G9a and mTOR activity (Refs 152, 153). An indole derivative of 3,3′-diindolylmethane also plays a regulatory role in gastric cancer and inhibits miR-30e leading to ATG5 activation (Ref. 154). Honokiol is a lignan isolated from the genus *Magnolia* L. (Magnoliaceae) that inhibits melanoma stem cells by targeting notch signalling and induced in autophagic cell death (Ref. 155). Paeoniflorigenone regulated autophagy to induce anticancer bioactivities in human head and neck squamous cell carcinomas. This compound inactivated PI3K/AKT/mTOR/p70S6K in YD-10B cells (Ref. 156). *β*-lapachone and plumbagin, the active naphthoquinone, showed potent anticancer effects through autophagy induction (Refs 157, 158). *p*-Coumaric acid, an ubiquitous plant phenolic acid, had cytotoxicity on neuroblastoma via generation of ROS that enhanced autophagy-induced mitochondria dysfunction (Ref. 159). Elaiophylin, a natural autophagy inhibitor, exerted antitumour activity in multiple myeloma and ovarian cancer (Refs 70, 160). Polyphenols are a large group of natural compounds that played an important role in modulating autophagy. They had significant antioxidant and anti-inflammatory properties as well as autophagic regulation in cancer cells. Curcumin is a natural polyphenol derived from rhizomes of *Curcuma longa* L. (Zingiberaceae) commonly known as turmeric. Numerous studies indicated that curcumin was able to modulate autophagy against various cancer including prostate, colon, oral, pancreatic cancers and brain tumours (Refs 161, 162, 163, 164, 165, 166). Withaferin A had a steroidal lactone structure and inhibited lysosomal activity to block autophagic flux in breast cancer cells (Ref. 167). Periplocin is a natural active steroid isolated from *Periploca forrestii* Schltr. (Apocynaceae). This compound promoted autophagy in pancreatic cancer cells via regulating the AMPK/mTOR pathway (Ref. 168). Resveratrol (3,5,4-trihydroxystilbene) is a stilbenoid found mainly in red grape, cranberry, mulberry, and peanut. It has gained more attention over the past two decades because of its ability to prevent and treat various cancers (Ref. 169). A link between resveratrol and autophagy regulation by different moleculer mechanism was reported in breast, colon and ovarian cancers (Refs 170, 171, 172). Corilagin and punicalagin, members of the tannin group, induced autophagy in gastric cancer and glioblastoma cell lines, respectively (Refs 173, 174). Lucidone, a terpeneoid, inhibited autophagy via HMGB1/RAGE/PI3K/Akt signalling pathway in pancreatic cancer cells (Ref. 175). Celastrol induced autophagy by targeting AR/miR-101 and served as epigenetic regulator in prostate cancer cell lines (Ref. 176). Two natural triterpenoids, toosendanin and isotoosendanin, suppressed triple-negative breast cancer growth through inducing autophagy (Ref. 177).

### Autophagic mechanisms of natural compounds in cardiac diseases

**Figure 4.**
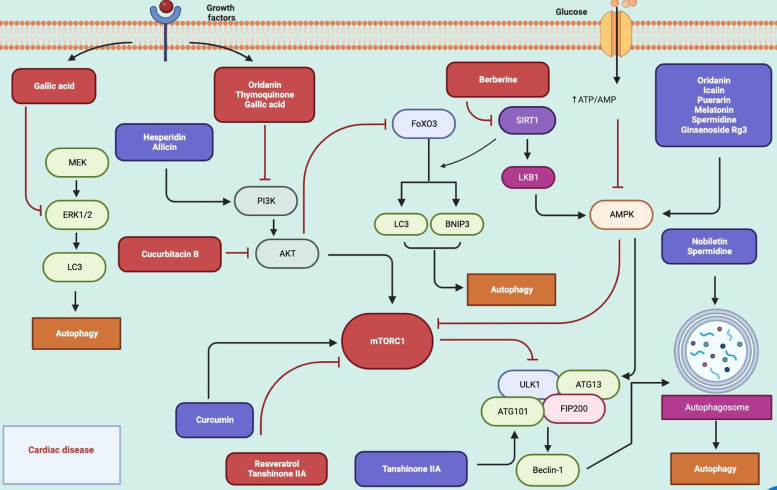
Autophagy-related pathways regulated by natural compounds in cardiac disease (Figure created with Biorender.com).

Despite recent advances in therapeutic regimens, cardiac disease is still a leading cause of morbidity and mortality and remains one of the greatest threats to public health. Dysregulation of autophagy in cardiomyocytes is associated with myocardial infarction, cardiac hypertrophy, heart failure and diabetic cardiomyopathy. In this context, autophagy appears to be an important therapeutic target and delays cardiac aging (Ref. 178). Different groups of natural compounds including berberine, tanshinone IIA, oridanin, hesperidin, icariin, luteolin, nobiletin, puerarin, melatonin, hinokitiol, thymoquinone, gallic acid, spermidine, curcumin, allicin, resveratrol, ginsenoside Rg3, cucurbitacin B were reported to display cardioprotective effect in experimental animal models and *in vitro* studies via autophagy modulation (Refs 179, 180, 181, 182, 183, 184, 185, 186, 187, 188, 189, 190, 191, 192, 193, 194, 195, 196, 197, 198, 199, 200). Autophagy regulating natural compounds in cardiac diseases and molecular target mechanisms were summarised in Table 6 and Figure 4.

**Table 6.** Autophagy regulating natural compounds in cardiac diseases

Group	Compound name	*in vitro/in vivo*	Model	Autophagic target	Molecular target	References
Alkaloids	Berberine	*in vivo*	Myocardial ischaemia/reperfusion (I/R)-induced injury	Autophagy inhibition	Decreases SIRT1, BNIP3, Beclin-1	(Ref. 179)
Alkaloids	Berberine	*in vitro*	Hypoxia/reoxygenisation with H9c2 cells	Autophagy inhibition	Decreases p-AMPK and p-mTORC2	(Ref. 179)
Alkaloids	Berberine	*in vivo*	Constriction of abdominal aorta (CAA)-induced yyocardial hypertrophy	Autophagy induction	Reduces LncRNA MIAT expression	(Ref. 180)
Diterpenoids	Tanshinone IIA	*in vivo*	Doxorubicin (DOX)-induced cardiotoxicity	Autophagy induction	Inhibits mTOR; activate ULK1-Beclin-1/TFEB-LAMP1	(Ref. 181)
Diterpenoids	Oridanin	*in vivo*	Aortic banding (AB)-induced cardiac hypertrophy	Autophagy induction	Inhibits Akt/mTORC1, activate AMPK and p21	(Ref. 182)
Flavonoids	Hesperidin	*in vivo*	Myocardial I/R-induced injury	Autophagy inhibition	Activates PI3K/Akt/mTOR pathway	(Ref. 183)
Flavonoids	Icariin	*in vitro*	ISO-induced H9c2 or NRCM myocytes hypertrophy	Autophagy induction	Mediates AMPK/mTOR pathway	(Ref. 184)
Flavonoids	Luteolin	*in vivo*	Left anterior descending (LAD)-ligation induced myocardial infarction	Autophagy induction	Inhibits Mst1	(Ref. 185)
Flavonoids	Nobiletin	*in vivo*	LAD-ligation induced myocardial infarction	Autophagy inhibition	Increases autophagosome conversion to autophagolysosome	(Ref. 186)
Flavonoids	Puerarin	*in vivo*	AB-induced cardiac hypertrophy	Autophagy induction	Activates AMPK/mTOR/S6K	(Ref. 187)
Indoleamines	Melatonin	*in vivo*	Myocardial I/R-induced injury	Autophagy inhibition	Activates AMPK/mTOR	(Ref. 188)
Indoleamines	Melatonin	*in vitro*	I/R injury with neonatal CMECs	Autophagy inhibition	Activates AMPK/mTOR	(Ref. 189)
Indoleamines	Melatonin	*in vivo*	Chronic intermittent hypoxia (CIH)-induced cardiac hypertrophy	Autophagy induction	Activates AMPK	(Ref. 189)
Monoterpenoids	Hinokitiol	*in vitro*	H_2_O_2_-induced oxidative damaged in cardiomyocytes	Autophagy inhibition	Inhibits GSK3*β*	(Ref. 190)
Monoterpenoids	Thymoquinone	*in vivo*	Sepsis-induced cardiac damage	Autophagy induction	Inhibits PI3K	(Ref. 191)
Phenolic acids	Gallic acid	*in vivo*	Transverse aortic constrictio (TAC)-induced cardiac hypertrophy and heart failure	Autophagy induction	Inhibits Akt, ERK1/2, ULK1	(Ref. 192)
Polyamines	Spermidine	*in vivo*	High-salt-induced congestive heart failure	Autophagy induction	Enhances autophagic flux	(Ref. 193)
Polyamines	Spermidine	*in vivo*	Myocardial infarction-induced myocardial injury	Autophagy induction	Promotes AMPK/mTOR	(Ref. 194)
Polyphenolics	Curcumin	*in vitro*	Isoproterenol (ISO)-induced cardiac hypertrophy and fibrosis	Autophagy inhibition	Activates mTOR	(Ref. 195)
Senevol glycosides	Allicin	*in vivo*	Pressure overload-induced cardiac hypertrophy	Autophagy inhibition	Activates PI3K/Akt/mTOR and MAPK/ERK/mTOR	(Ref. 196)
Stilbenes	Resveratrol	*in vivo*	DOX-induced cardiotoxicity	Autophagy induction	Inhibits E2F1/mTORC1, E2F1/AMPK*α*2	(Ref. 197)
Stilbenes	Resveratrol	*in vitro*	DOX-induced cardiomyocyte death	Autophagy induction	Inhibits S6K1	(Ref. 198)
Triterpenoid saponins	Ginsenoside Rg3	*in vivo*	ISO-induced myocardial infarction	Autophagy induction	Activates AMPK downstream signalling pathway	(Ref. 199)
Triterpenoids	Cucurbitacin B	*in vivo*	Aortic banding (AB)-induced cardiac hypertrophy	Autophagy induction	Inhibits Akt/mTOR/FoXO3a	(Ref. 200)

### Autophagic mechanisms of natural compounds in neurological aging

**Figure 5.**
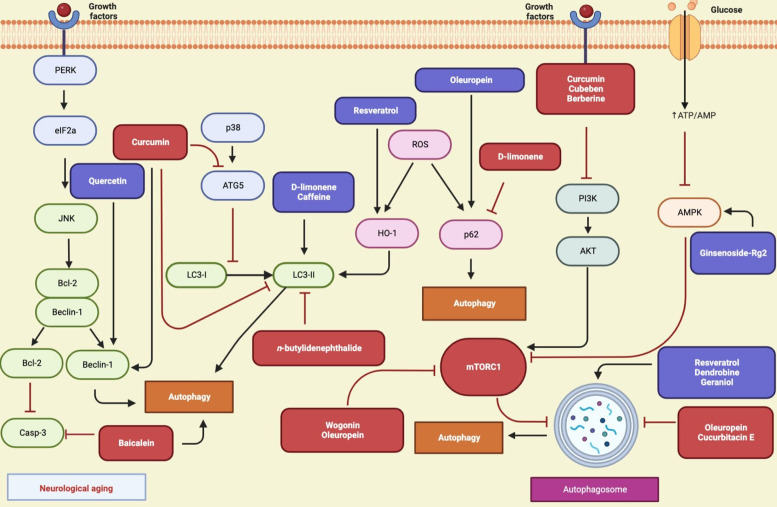
Autophagy-related pathways regulated by natural compounds in neurological aging (Figure created with Biorender.com).

Many studies indicated that natural compounds had therapeutic benefits in NDs via different mechanisms by targeting autophagy. Autophagy regulating natural compounds in neurological aging and molecular target mechanisms were given in Table 7 and Figure 5. Berberine, conophylline, curcumin, ginsenoside-Rg2, and celastrol improved cognitive impairment via autophagy induction in mice models of AD (Refs 201, 202, 203, 204, 205). Caffeine is a well-known natural compound commonly used to increase alertness and energy. It prevented human prion protein-mediated neurotoxicity via autophagy induction (Ref. 206). Dendrobine, an alkaloid, induced autophagy flux in hippocampus primary neurons of rats (Ref. 207). Abnormalities in neuron axonal transport-related proteins inhibit autophagosome maturation in AD. Curcumin increased autophagic flux by inducing interactions among autophagic axonal transport-related proteins and promoting lysosome-autophagosome fusion in N2a/APP695swe cells (Ref. 208). Curcumin also showed a protective effect on amyloid-*β*-42 induced cytotoxicity in HT-22 cells (Ref. 209). *β*-asarone and cubeben inhibited amyloid-*β*, and PI3K-AKT, respectively via promoting autophagy in AD model with neuronal cells (Refs 210, 211). Wogonin, natural active flavonoid, increased *β*-amyloid clearance and inhibited mTOR in SH-SHY5Y cells (Ref. 212). Baicalein inhibited caspase-3 activity and exhibited beneficial effect in PD model with SH-SY5Y (Ref. 213). Calycosin, an phytoestrogen, showed protective effect against paraquat (PQ)-induced neurodegeneration by reducing pS6K and p4EBP1 levels (Ref. 214). Quercetin, a flavonoid known for its neuroprotective effects, acted as an autophagy enhancer and upregulated Beclin-1 in PD rat model (Ref. 215). *n*-Butylidenephthalide induced autophagic down-regulation in motor neurons and prolonged the survival of amyotrophic lateral sclerosis (ALS) mice (Ref. 216). Oleuropein is the active constituent of olive leaves and fruits and known as antioxidant, neuroprotective and autophagy-regulating properties. These polyphenolic compounds showed therapeutic benefits against PD in neuronal PC12 cells and TgCRND8 mouse model (Refs 217, 218). Resveratrol was reported to have neuroprotective potential in HD. It protected mutant SH-SY5Y cells from dopamine toxicity via rescuing ATG4-mediated mediated autophagosome formation (Ref. 219). Also, resveratrol showed beneficial effects by inducing autophagy in both *in vitro* and *in vivo* AD and PD models (Refs 220, 221, 222). D-limonene is a fragrance agent and belongs to the terpene group. D-limonene increased LC3-II and reduced p62 levels by inducing autophagy in SH-SY5Y cells (Ref. 223). Cucurbitacin E is a tetracyclic triterpenoid isolated from Cucurbitaceae plants, decreased autophagic flux and neuronal death in a postmitotic cellular model of PD (Ref. 224). Geraniol also diminished autophagy flux in an *in vitro* model of PD (Ref. 225). Cannabidiol, a natural compound from *Cannabis sativa* L. (Cannabaceae) extended lifespan and improved neuronal health in *C. elegans* by induction of autophagy (Ref. 226).

**Table 7.** Autophagy regulating natural compounds in neurological aging

Group	Compound name	*in vitro/in vivo*	Model	Autophagic target	Molecular target	References
Alkaloids	Berberine	*in vivo*	APP/tau/PS1 mouse model of AD	Autophagy induction	Induces the class III PI3K/Beclin-1 pathway	(Ref. 201)
Alkaloids	Caffeine	*in vitro*	Prion-mediated neurotoxicity with SH-SY5Y	Autophagy induction	Increaes LC3-II	(Ref. 206)
Alkaloids	Conophylline	*in vitro*	AD and HD model with PC12	Autophagy induction	Increases protein aggregation	(Ref. 202)
Alkaloids	Dendrobine	*in vivo*	Rat model of AD	Autophagy induction	Increases autophagic flux	(Ref. 207)
Alkaloids	*n*-butylidenephthalide	*in vivo*	SOD1-G93A mouse model of Amyotrophic lateral sclerosis (ALS)	Autophagy inhibition	Inhibits LC3-II and caspase 3	(Ref. 216)
Cannabinoids	Cannabidiol	*in vivo*	Neuronal aging model in *Caenorhabditis elegans*	Autophagy induction	Modulates SIRT1/sir-2.1	(Ref. 226)
Flavonoids	Baicalein	*in vitro*	PD model with SH-SY5Y	Autophagy induction	Inhibits caspase-3 activity	(Ref. 213)
Flavonoids	Calycosin	*in vivo*	Paraquat (PQ)-induced PD model in *Drosophila*	Autophagy induction	Reduces pS6K and p4EBP1 levels	(Ref. 214)
Flavonoids	Quercetin	*in vivo*	Rotenone rat model of PD	Autophagy induction	Upregulates Beclin-1 expression	(Ref. 215)
Flavonoids	Wogonin	*in vitro*	SH-SY5Y, SH-SHY5Y cells	Autophagy induction	Inhibits mTOR	(Ref. 212)
Phenylpropanoids	*β*-Asarone	*in vitro*	AD model with PC12	Autophagy induction	Inhibits amyloid-*β*	(Ref. 210)
Polyphenols	Curcumin	*in vivo*	APP/PS1 mouse model of AD	Autophagy induction	Downregulates PI3K/Akt/mTOR	(Ref. 203)
Polyphenols	Curcumin	*in vitro*	Mouse hippocampal neuronal cell line (HT-22)	Autophagy inhibition	Downregulates Beclin-1	(Ref. 209)
Polyphenols	Curcumin	*in vitro*	AD model with N2a/APP695swe	Autophagy induction	Increases Beclin-1, ATG5, and ATG16L1	(Ref. 208)
Polyphenols	Oleuropein	*in vitro*	PD model with PC12	Autophagy modulation	Inhibits autophagic flux and increases p62	(Ref. 217)
Polyphenols	Oleuropein	*in vivo*	TgCRND8 PD mouse model	Autophagy induction	Inhibits mTOR	(Ref. 218)
Silbenes	Resveratrol	*in vitro*	HD model with SH-SY5Y	Autophagy induction	Induces autophagosomes formation	(Ref. 219)
Silbenes	Resveratrol	*in vitro*	PD model with SH-SY5Y	Autophagy induction	Increases HO-1 expression and autophagic flux	(Ref. 220)
Silbenes	Resveratrol	*in vitro*	AD model with PC12	Autophagy induction	Activates the TyrRS-PARP1-SIRT1	(Ref. 221)
Silbenes	Resveratrol	*in vivo*	MPTP 1-methyl-4-phenyl-1,2,3,6-tetrahydropyridine (MPTP)-induced PD mouse model	Autophagy induction	Avtivates SIRT1-deacetylated LC3	(Ref. 222)
Terpenes	Cubeben	*in vitro*	AD model with neuronal cells	Autophagy induction	Inhibits PI3K-AKT	(Ref. 211)
Terpenes	D-Limonene	*in vitro*	SH-SY5Y	Autophagy induction	Increases LC3-II and reduces p62 levels	(Ref. 223)
Terpenes	Geraniol	*in vitro*	PD model with SK-N-SH	Autophagy inhibition	Diminishes autophagy flux	(Ref. 225)
Triterpenoid saponins	Ginsenoside-Rg2	*in vivo*	5XFAD mouse model of AD	Autophagy induction	AMPK-ULK1-dependent and mTOR-independent	(Ref. 204)
Triterpenoids	Celastrol	*in vivo*	P301S Tau and 3xTg transgenic AD mouse model	Autophagy induction	Activates TFEB	(Ref. 205)
Triterpenoids	Cucurbitacin E	*in vitro*	PD model with PC12	Autophagy inhibition	Maintaines lysosomal distribution and decreases autophagy flux	(Ref. 224)

### Autophagic mechanisms of natural compounds in obesity and diabetes

**Figure 6.**
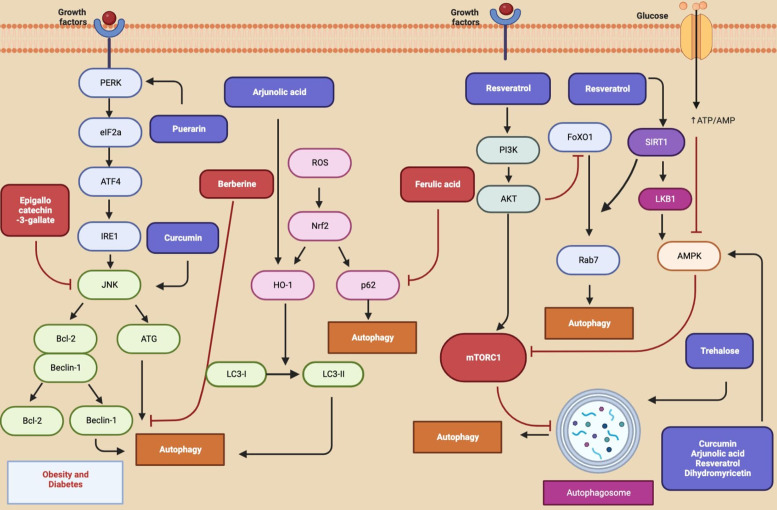
Autophagy-related pathways regulated by natural compounds in obesity and diabetes (Figure created with Biorender.com).

Autophagy has a modulating role in the process of adipocyte conversion. It is also an important signalling pathway for T2DM. Autophagy regulating natural compounds in obesity and diabetes and molecular target mechanisms were given in Table 8 and Figure 6. Berberine, an alkaloid, inhibited basal autophagy in adipocytes in mice fed a high-fat diet by decreasing Beclin-1 (Ref. 227). Resveratrol increased AMPK and improved health and survival of high-calorie diet induced mice (Ref. 228). Resveratrol activated SIRT1/FoXO1/Rab7 and ameliorated myocardial oxidative stress injury in diabetic mice (Ref. 229). It alleviated I/R injury of diabetic myocardium by promoting autophagy (Ref. 230). Apart from resveratrol, epigallocatechin-3-gallate, trehalose, dihydromyricetin, puerarin, melatonin, ferulic acid, arjunolic acid, and curcumin were reported to effective in experimental animal models and *in vitro* studies of diabetes via autophagy modulation (Refs 231, 232, 233, 234, 235, 236, 237, 238, 239).

**Table 8.** Autophagy regulating natural compounds in obesity and diabetes

Group	Compound name	*in vitro/in vivo*	Model	Autophagic target	Molecular target	References
Alkaloids	Berberine	*in vivo*	High-fat diet induced obesity	Autophagy inhibition	Decreases Beclin-1	(Ref. 227)
Catechins	Epigallocatechin-3-gallate	*in vivo*	T2DM-induced damage in skeletal muscle	Autophagy inhibition	Regulates JNK	(Ref. 231)
Disaccharides	Trehalose	*in vitro*	Hyperglycaemia model in neuroepithelial cells	Autophagy induction	Induces autophagosome formation.	(Ref. 232)
Flavonoids	Dihydromyricetin	*in vivo*	Insulin sensitivity mouse model	Autophagy induction	Activates AMPK-PGC-1*α*-Sirt3	(Ref. 233)
Flavonoids	Puerarin	*in vivo*	STZ-induced diabetic nephropathy	Autophagy induction	Regulates PERK/eIF2*α*/ATF4	(Ref. 234)
Indoleamines	Melatonin	*in vivo*	STZ-induced diabetic cardiomyopathy	Autophagy induction	Inhibit Mst1/Sirt3 pathway	(Ref. 235)
Phenolic acids	Ferulic acid	*in vivo*	STZ/HFD-induced diabetic nephropathy	Autophagy induction	Decreases p62 expression	(Ref. 236)
Phenolics	Arjunolic acid	*in vivo*	STZ-induced diabetic retinopathy	Autophagy induction	Activates AMPK/mTOR/HO-1	(Ref. 237)
Polyphenolics	Curcumin	*in vivo*	STZ-induced diabetic cardiomyopathy	Autophagy induction	Activates AMPK/mTOR and JNK1	(Ref. 238)
Polyphenolics	Curcumin	*in vivo*	STZ-induced diabetic nephropathy	Autophagy inhibition	Regulates Beclin-1/UVRAG/Bcl2	(Ref. 239)
Stilbenes	Resveratrol	*in vivo*	High-calorie diet induced obesity	Autophagy induction	Increases AMPK	(Ref. 228)
Stilbenes	Resveratrol	*in vivo*	Myocardial oxidative stress injury model in diabetic mice	Autophagy inhibition	Activates SIRT1/FoXO1/Rab7	(Ref. 229)
Stilbenes	Resveratrol	*in vivo*	I/R-induced injury of diabetic myocardium	Autophagy induction	Activate PI3K	(Ref. 230)

## Conclusion and future perspectives

Aging, an irreversible biological process, is an independent risk factor for cancer, neurodegeneration, cardiovascular diseases, obesity and diabetes. Under both healthy and pathological circumstances, autophagy is a crucial mechanism. Studies have revealed that the dysregulation of autophagy plays a role in the pathogenesis of illnesses associated with aging and have proposed potential treatments involving the control of the autophagy system. It is thought that a number of bioactive substances generated from medicinal plants have the ability to regulate autophagy by concentrating on autophagic pathways. The targeted control of autophagy is thought to offer a method of treating age-related disorders, hence regulation of the autophagic process is increasingly seen as an intriguing drug development technique. The regulating function of natural substances on autophagy to delay or treat age-related illnesses *in vitro* and in animal models was the main focus of this review. However, there is broad agreement about the effectiveness of natural pleiotropic substances capable of enhancing or reestablishing deficient autophagy in aggregate-prone proteins. According to literature data, different groups of phytochemicals have attracted attention as promising autophagy modulators. Curcumin and resveratrol stand out in terms of effectiveness and popularity and these two compounds can be identified as potent autophagy regulating compounds in cancer, cardiovascular and NDs, and metabolic dysfunction. Keep in mind that autophagy can be a two-way process. To successfully combat age-related illnesses, significant improvements in our understanding of their modes of action, pharmacokinetics, and nonspecific effects are required.

## References

[1] Aman Y (2021) Autophagy in healthy aging and disease. Nature Aging 1, 634–650. 34901876 10.1038/s43587-021-00098-4 PMC8659158

[2] Menzies FM (2017) Autophagy and neurodegeneration: pathogenic mechanisms and therapeutic opportunities. Neuron 93, 1015–1034. 28279350 10.1016/j.neuron.2017.01.022

[3] Ruckenstuhl C (2014) Lifespan extension by methionine restriction requires autophagy-dependent vacuolar acidification. PLoS Genetics 10, e1004347. 24785424 10.1371/journal.pgen.1004347 PMC4006742

[4] Gatica D (2015) Molecular mechanisms of autophagy in the cardiovascular system. Circulation Research 116, 456–467. 25634969 10.1161/CIRCRESAHA.114.303788 PMC4313620

[5] Tan CC (2014) Autophagy in aging and neurodegenerative diseases: implications for pathogenesis and therapy. Neurobiology of Aging 35, 941–957. 24360503 10.1016/j.neurobiolaging.2013.11.019

[6] Jaishy B (2015) Lipid-induced NOX2 activation inhibits autophagic flux by impairing lysosomal enzyme activity. Journal of Lipid Research 56, 546–561. 25529920 10.1194/jlr.M055152 PMC4340303

[7] Rahman MA (2020) Molecular insights into the multifunctional role of natural compounds: autophagy modulation and cancer prevention. Biomedicines 8, 517. 33228222 10.3390/biomedicines8110517 PMC7699596

[8] Majeski AE (2004) Mechanisms of chaperone-mediated autophagy. International Journal of Biochemistry & Cell Biology 36, 2435–2444. 15325583 10.1016/j.biocel.2004.02.013

[9] Kroemer G (2010) Autophagy and integrated stress response. Molecular Cell 40, 280–293. 20965422 10.1016/j.molcel.2010.09.023 PMC3127250

[10] Tekirdag K and Cuervo AM (2018) Chaperone-mediated autophagy and endosomal microautophagy: jointed by a chaperone. Journal of Biological Chemistry 293, 5414–5424. 29247007 10.1074/jbc.R117.818237 PMC5900761

[11] Vitale E (2022) Apelin-13 increases functional connexin-43 through autophagy inhibition via AKT/mTOR pathway in the non-myocytic cell population of the heart. International Journal of Molecular Sciences 23, 13073. 36361860 10.3390/ijms232113073 PMC9655608

[12] Huang J (2007) Autophagy and human disease. Cell Cycle 6, 1837–1849. 17671424 10.4161/cc.6.15.4511

[13] Klionsky DJ (2003) A unified nomenclature for yeast autophagy-related genes. Developmental 5, 539–545. 10.1016/s1534-5807(03)00296-x 14536056

[14] Kovacs AL (2010) Role of autophagy in *Caenorhabditis elegans*. FEBS Letters 584, 1335–1341. 20138173 10.1016/j.febslet.2010.02.002

[15] Karampa AD (2022) The role of macroautophagy and chaperone-mediated autophagy in the pathogenesis and management of hepatocellular carcinoma. Cancers 14, 760. 35159028 10.3390/cancers14030760 PMC8833636

[16] Hu Y and Reggiori F (2022) Molecular regulation of autophagosome formation. Biochemical Society transactions 50, 55–69. 35076688 10.1042/BST20210819 PMC9022990

[17] Sanchez-Mirasierra I (2022) Targeting macroautophagy as a therapeutic opportunity to treat Parkinson's disease. Frontiers in Cell and Developmental Biology 10, 921314. 35874822 10.3389/fcell.2022.921314 PMC9298504

[18] Scrivo A (2018) Selective autophagy as a potential therapeutic target for neurodegenerative disorders. The Lancet. Neurology 17, 802–815. 30129476 10.1016/S1474-4422(18)30238-2 PMC6359907

[19] McCarty MF (2022) Nutraceutical and dietary strategies for up-regulating macroautophagy. International journal of molecular sciences 23, 2054. 35216170 10.3390/ijms23042054 PMC8875972

[20] Massey AC, Zhang C and Cuervo AM (2006) Chaperone-mediated autophagy in aging and disease. Current Topics in Developmental Biology 73, 205–235. 16782460 10.1016/S0070-2153(05)73007-6

[21] Arndt V (2010) Chaperone-assisted selective autophagy is essential for muscle maintenance. Current Biology 20, 143–148. 20060297 10.1016/j.cub.2009.11.022

[22] Lipinski MM (2010) Genome-wide analysis reveals mechanisms modulating autophagy in normal brain aging and in Alzheimer's disease. Proceedings of the National Academy of Sciences of the United States of America 107, 14164–9. 20660724 10.1073/pnas.1009485107 PMC2922576

[23] Fernández ÁF (2018) Disruption of the beclin 1-BCL2 autophagy regulatory complex promotes longevity in mice. Nature 558, 136–140. 29849149 10.1038/s41586-018-0162-7 PMC5992097

[24] Moreno-Blas D (2019) Cortical neurons develop a senescence-like phenotype promoted by dysfunctional autophagy. Aging (Albany NY) 11, 6175–6198. 31469660 10.18632/aging.102181 PMC6738425

[25] Nie D (2021) Rapamycin treatment of tendon stem/progenitor cells reduces cellular senescence by upregulating autophagy. Stem Cells International 2021, 6638249. 33603790 10.1155/2021/6638249 PMC7870298

[26] Cannizzo ES (2012) Age-related oxidative stress compromises endosomal proteostasis. Cell Reports 2, 136–149. 22840404 10.1016/j.celrep.2012.06.005 PMC3408590

[27] Valdor R (2014) Chaperone-mediated autophagy regulates T cell responses through targeted degradation of negative regulators of T cell activation. Nature Immunology 15, 1046–1054. 25263126 10.1038/ni.3003 PMC4208273

[28] Di Domenico F (2014) Oxidative stress and proteostasis network: Culprit and casualty of Alzheimer's-like neurodegeneration. Advances in Geriatrics 2014, 14.

[29] Um JH and Yun J (2017) Emerging role of mitophagy in human diseases and physiology. BMB Reports 50, 299–307. 28366191 10.5483/BMBRep.2017.50.6.056 PMC5498140

[30] Boya P, Codogno P and Rodriguez-Muela N (2018) Autophagy in stem cells: repair, remodelling and metabolic reprogramming. Development (Cambridge, England) 145, 1–14. 10.1242/dev.146506 29483129

[31] Rajendran P (2019) Autophagy and senescence: a new insight in selected human diseases. Journal of Cellular Physiology 234, 21485–21492. 31144309 10.1002/jcp.28895

[32] Hewitt G and Korolchuk VI (2017) Repair, reuse, recycle: the expanding role of autophagy in genome maintenance. Trends in Cell Biology 27, 340–351. 28011061 10.1016/j.tcb.2016.11.011

[33] Lopez-Otín C (2013) The hallmarks of aging. Cell 153, 1194–1217. 23746838 10.1016/j.cell.2013.05.039 PMC3836174

[34] Cheon SY (2019) Autophagy, cellular aging and age-related human diseases. Experimental Neurobiology 28, 643–657. 31902153 10.5607/en.2019.28.6.643 PMC6946111

[35] Fairlie WD, Tran S and Lee EF (2020) Crosstalk between apoptosis and autophagy signaling pathways. International Review of Cell and Molecular Biology 352, 115–158. 32334814 10.1016/bs.ircmb.2020.01.003

[36] Ge Y (2022) Role of AMPK mediated pathways in autophagy and aging. Biochimie 195, 100–113. 34838647 10.1016/j.biochi.2021.11.008

[37] Bhaskar PT and Hay N (2007) The two TORCs and Akt. Developmental Cell 12, 487–502. 17419990 10.1016/j.devcel.2007.03.020

[38] Papadopoli D (2019) mTOR as a central regulator of lifespan and aging. F1000Research 8, *F1000 Faculty Rev-998*, 1–21. 10.12688/f1000research.17196.1 PMC6611156 31316753

[39] Wang X (2020) AMPK and Akt/mTOR signalling pathways participate in glucose-mediated regulation of hepatitis B virus replication and cellular autophagy. Cellular Microbiology 22, e13131. 31746509 10.1111/cmi.13131

[40] Zada S (2021) Cross talk between autophagy and oncogenic signaling pathways and implications for cancer therapy. Biochimica et Biophysica Acta. Reviews on Cancer 1876, 188565. 33992723 10.1016/j.bbcan.2021.188565

[41] Chen Z (2019) Carvedilol exerts myocardial protection via regulation of AMPK-mTOR-dependent autophagy? Biomedicine & Pharmacotherapy = Biomedecine & Pharmacotherapie 118, 109283. 31376655 10.1016/j.biopha.2019.109283

[42] Pan X (2020) Accumulation of prelamin A induces premature aging through mTOR overactivation. FASEB Journal: Official Publication of the Federation of American Societies for Experimental Biology 34, 7905–7914. 32282093 10.1096/fj.201903048RR

[43] Zachari M and Ganley IG (2017) The mammalian ULK1 complex and autophagy initiation. Essays in Biochemistry 61, 585–596. 29233870 10.1042/EBC20170021 PMC5869855

[44] Turco E, Fracchiolla D and Martens S (2020) Recruitment and activation of the ULK1/Atg1 kinase complex in selective autophagy. Journal of Molecular Biology 432, 123–134. 31351898 10.1016/j.jmb.2019.07.027 PMC6971721

[45] Kitada M, Ogura Y and Koya D (2016) Role of Sirt1 as a Regulator of Autophagy, Autophagy: Cancer, Other Pathologies, Inflammation, Immunity, Infection, and Aging. Amsterdam, Netherlands: Academic Press, pp. 89–100.

[46] Lee IH (2008) A role for the NAD-dependent deacetylase Sirt1 in the regulation of autophagy. Proceedings of the National Academy of Sciences of the United States of America 105, 3374–3379. 18296641 10.1073/pnas.0712145105 PMC2265142

[47] Colman RJ (2009) Caloric restriction delays disease onset and mortality in *Rhesus* monkeys. Science (New York, N.Y.) 325, 201–204. 19590001 10.1126/science.1173635 PMC2812811

[48] Hansen M (2008) A role for autophagy in the extension of lifespan by dietary restriction in *C. elegans*. PLoS Genetics 4, e24. 18282106 10.1371/journal.pgen.0040024 PMC2242811

[49] Liu D (2019) PGC1*α* Activation by pterostilbene ameliorates acute doxorubicin cardiotoxicity by reducing oxidative stress via enhancing AMPK and SIRT1 cascades. Aging 11, 10061–10073. 31733141 10.18632/aging.102418 PMC6914429

[50] Fahy GM (2019) Reversal of epigenetic aging and immunosenescent trends in humans. Aging cell 18, e13028. 31496122 10.1111/acel.13028 PMC6826138

[51] Ao X, Zou L and Wu Y (2014) Regulation of autophagy by the Rab GTPase network. Cell Death and Differentiation 21, 348–358. 24440914 10.1038/cdd.2013.187 PMC3921601

[52] Salminen A and Kaarniranta K (2009) Regulation of the aging process by autophagy. Trends in Molecular Medicine 15, 217–224. 19380253 10.1016/j.molmed.2009.03.004

[53] Mak T (2003) The E. Donnall Thomas lecture-apoptosis: “Tis death that makes life live”. Biology of Blood and Marrow Transplantation 9, 483–488. 12931116 10.1016/s1083-8791(03)00215-5

[54] Anding AL and Baehrecke EH (2015) Autophagy in cell life and cell death. Current Topics in Developmental Biology 114, 67–91. 26431564 10.1016/bs.ctdb.2015.07.012

[55] Yu L, Chen Y and Tooze SA (2018) Autophagy pathway: cellular and molecular mechanisms. Autophagy 14, 207–215. 28933638 10.1080/15548627.2017.1378838 PMC5902171

[56] Ding WX, Chen X and Yin XM (2012) Tumor cells can evade dependence on autophagy through adaptation. Biochemical and Biophysical Research Communications 425, 684–688. 22842577 10.1016/j.bbrc.2012.07.090 PMC3432502

[57] Maycotte P and Thorburn A (2011) Autophagy and cancer therapy. Cancer Biology & Therapy 11, 127–137. 21178393 10.4161/cbt.11.2.14627 PMC3047083

[58] Alers S (2012) Role of AMPK-mTOR-Ulk1/2 in the regulation of autophagy: cross talk, shortcuts, and feedbacks. Molecular and Cellular Biology 32, 2–11. 22025673 10.1128/MCB.06159-11 PMC3255710

[59] Liang XH (1999) Induction of autophagy and inhibition of tumorigenesis by beclin 1. Nature 402, 672–676. 10604474 10.1038/45257

[60] Mathew R, Karantza-Wadsworth V and White E (2007) Role of autophagy in cancer. Nature Reviews Cancer 7, 961–967. 17972889 10.1038/nrc2254 PMC2866167

[61] Miracco C (2007) Protein and mRNA expression of autophagy gene Beclin 1 in human brain tumours. International Journal of Oncology 30, 429–436. 17203225

[62] Liang C (2006) Autophagic and tumour suppressor activity of a novel Beclin1-binding protein UVRAG. Nature Cell Biology 8, 688–699. 16799551 10.1038/ncb1426

[63] Salvi A (2022) PHY34 inhibits autophagy through V-ATPase V0A2 subunit inhibition and CAS/CSE1L nuclear cargo trafficking in high grade serous ovarian cancer. Cell Death and Disease 13, 45. 35013112 10.1038/s41419-021-04495-w PMC8748433

[64] Huang W (2022) UBE2T is upregulated, predicts poor prognosis, and promotes cell proliferation and invasion by promoting epithelial-mesenchymal transition via inhibiting autophagy in an AKT/mTOR dependent manner in ovarian cancer. Cell Cycle 21, 780–791. 35130130 10.1080/15384101.2022.2031426 PMC8973388

[65] Rao RD, Buckner JC and Sarkaria JN (2004) Mammalian target of rapamycin (mTOR) inhibitors as anticancer agents. Current Cancer Drug Targets 4, 621–635. 15578919 10.2174/1568009043332718

[66] Zaytseva YY (2012) mTOR inhibitors in cancer therapy. Cancer Letters 319, 1–7. 22261336 10.1016/j.canlet.2012.01.005

[67] Motzer RJ (2008) RECORD-1 Study group. Efficacy of everolimus in advanced renal cell carcinoma: a double-blind, randomised, placebo-controlled phase III trial. Lancet 372, 449–456. 18653228 10.1016/S0140-6736(08)61039-9

[68] Jurczak W (2018) Comparison of two doses of intravenous temsirolimus in patients with relapsed/refractory mantle cell lymphoma. Leukemia & Lymphoma 59, 670–678. 28768446 10.1080/10428194.2017.1357175

[69] Yao JC (2008) Efficacy of RAD001 (everolimus) and octreotide LAR in advanced low- to intermediate-grade neuroendocrine tumors: results of a phase II study. Journal of Clinical Oncology 10, 4311–4318. 10.1200/JCO.2008.16.7858 PMC2653122 18779618

[70] Zhao X (2015) Elaiophylin, a novel autophagy inhibitor, exerts antitumor activity as a single agent in ovarian cancer cells. Autophagy 11, 1849–1863. 25893854 10.1080/15548627.2015.1017185 PMC4824600

[71] Liang DH, El-Zein R and Dave B (2015) Autophagy inhibition to increase radiosensitization in breast cancer. Journal of Nuclear Medicine & Radiation Therapy 6, 254. 26613064 10.4172/2155-9619.1000254 PMC4657142

[72] Barnard RA (2014) Phase I clinical trial and pharmacodynamic evaluation of combination hydroxychloroquine and doxorubicin treatment in pet dogs treated for spontaneously occurring lymphoma. Autophagy 10, 1415–1425. 24991836 10.4161/auto.29165 PMC4203518

[73] Malet-Martino M, Jolimaitre P and Martino R (2022) The prodrugs of 5-fluorouracil. Current Medicinal Chemistry – Anti-Cancer Agents 2, 267–310. 10.2174/1568011023354146 12678747

[74] Liang X (2014) Suppression of autophagy by chloroquine sensitizes 5-fluorouracil-mediated cell death in gallbladder carcinoma cells. Cell & Bioscience 4, 10. 24581180 10.1186/2045-3701-4-10 PMC4015784

[75] Kunimasa K (2022) Spautin-1 inhibits mitochondrial complex I and leads to suppression of the unfolded protein response and cell survival during glucose starvation. Scientific Reports 12, 11533. 35798783 10.1038/s41598-022-15673-x PMC9262966

[76] Zhao XG (2015) Chloroquine-enhanced efficacy of cisplatin in the treatment of hypopharyngeal carcinoma in xenograft mice. PLoS ONE 10, e0126147. 25923669 10.1371/journal.pone.0126147 PMC4414471

[77] Shirakabe A (2016) Aging and autophagy in the heart. Circulation Research 118, 1563–1576. 27174950 10.1161/CIRCRESAHA.116.307474 PMC4869999

[78] Zhang H (2008) Mitochondrial autophagy is an HIF-1-dependent adaptive metabolic response to hypoxia. Journal of Biological Chemistry 283, 10892–10903. 18281291 10.1074/jbc.M800102200 PMC2447655

[79] Matsui Y (2007) Distinct roles of autophagy in the heart during ischemia and reperfusion: roles of AMP-activated protein kinase and Beclin 1 in mediating autophagy. Circulation Research 30, 914–922. 10.1161/01.RES.0000261924.76669.36 17332429

[80] Hao M (2017) Myocardial ischemic postconditioning promotes autophagy against ischemia reperfusion injury via the activation of the nNOS/AMPK/mTOR pathway. International Journal of Molecular Sciences 18, 614. 28287478 10.3390/ijms18030614 PMC5372630

[81] Dämmrich J and Pfeifer U (1983) Cardiac hypertrophy in rats after supravalvular aortic constriction. II. Inhibition of cellular autophagy in hypertrophying cardiomyocytes. Virchows Archiv. B, Cell Pathology Including Molecular Pathology 43, 287–307. 6137901 10.1007/BF02932962

[82] Nakai A (2007) The of autophagy in cardiomyocytes in the basal state and in response to hemodynamic stress. Nature Medicine 3, 619–624. 10.1038/nm1574 17450150

[83] Nemchenko A (2011) Autophagy as a therapeutic target in cardiovascular disease. Journal of Molecular and Cellular Cardiology 51, 584–593. 21723289 10.1016/j.yjmcc.2011.06.010 PMC3177001

[84] Hansen M, Rubinsztein DC and Walker DW (2018) Autophagy as a promoter of longevity: insights from model organisms. Nature Reviews. Molecular Cell Biology 19, 579–593. 30006559 10.1038/s41580-018-0033-y PMC6424591

[85] Holtzman DM, Morris JC and Goate AM (2011) Alzheimer's disease: the challenge of the second century. Science Translational Medicine 3, 1–35. 10.1126/scitranslmed.3002369 PMC3130546 21471435

[86] Halliday G, Lees A and Stern M (2011) Milestones in Parkinson's disease-clinical and pathologic features. Movement Disorders: Official Journal of the Movement Disorder Society 26, 1015–1021. 21626546 10.1002/mds.23669

[87] Senkevich K and Gan-Or Z (2020) Autophagy lysosomal pathway dysfunction in Parkinson's disease; evidence from human genetics. Parkinsonism & Related Disorders 73, 60–71. 31761667 10.1016/j.parkreldis.2019.11.015

[88] Pickrell AM and Youle RJ (2015) The roles of PINK1, Parkin and mitochondrial fidelity in Parkinson's disease. Neuron 85, 257–273. 25611507 10.1016/j.neuron.2014.12.007 PMC4764997

[89] Recchia A (2004) α-Synuclein and Parkinson's disease. The FASEB Journal 18, 617–626. 15054084 10.1096/fj.03-0338rev

[90] Winslow AR (2010) *α*-Synuclein impairs macroautophagy: implications for Parkinson's disease. The Journal of Cell Biology 190, 1023–1037. 20855506 10.1083/jcb.201003122 PMC3101586

[91] McColgan P and Tabrizi SJ (2018) Huntington's disease: a clinical review. European Journal of Neurology 25, 24–34. 28817209 10.1111/ene.13413

[92] Jeong H (2009) Acetylation targets mutant huntingtin to autophagosomes for degradation. Cell 137, 72. 10.1016/j.cell.2009.03.018 PMC2940108 19345187

[93] Wu JC (2012) The regulation of N-terminal Huntingtin (Htt552) accumulation by Beclin1. Acta Pharmacologica Sinica 33, 751. 10.1038/aps.2012.14 PMC4010368 22543707

[94] Fu Y, Sun X and Lu B (2018) HIPK3 modulates autophagy and HTT protein levels in neuronal and mouse models of Huntington disease. Autophagy 14, 169–170. 29130397 10.1080/15548627.2017.1393130 PMC5846548

[95] Walter C (2016) Activation of AMPK-induced autophagy ameliorates Huntington disease pathology *in vitro*. Neuropharmacology 108, 24–38. 27133377 10.1016/j.neuropharm.2016.04.041

[96] Pickford F (2008) The autophagy-related protein beclin 1 shows reduced expression in early Alzheimer disease and regulates amyloid beta accumulation in mice. The Journal of Clinical Investigation 118, 2190–2199. 18497889 10.1172/JCI33585 PMC2391284

[97] Khandelwal PJ (2011) Parkin mediates beclin-dependent autophagic clearance of defective mitochondria and ubiquitinated Abeta in AD models. Human Molecular Genetics 20, 2091–2102. 21378096 10.1093/hmg/ddr091 PMC3090189

[98] Rocchi A (2017) A Becn1 mutation mediates hyperactive autophagic sequestration of amyloid oligomers and improved cognition in Alzheimer's disease. PLoS Genetics 13, e1006962. 28806762 10.1371/journal.pgen.1006962 PMC5570506

[99] Ma Q (2011) Age-related autophagy alterations in the brain of senescence accelerated mouse prone 8 (SAMP8) mice. Experimental Gerontology 46, 533–541. 21385605 10.1016/j.exger.2011.02.006

[100] Caccamo A (2013) mTOR regulates tau phosphorylation and degradation: implications for Alzheimer's disease and other tauopathies. Aging Cell 12, 370–380. 23425014 10.1111/acel.12057 PMC3655115

[101] Caccamo A (2011) Naturally secreted amyloid-beta increases mammalian target of rapamycin (mTOR) activity via a PRAS40-mediated mechanism. The Journal of Biological Chemistry 286, 8924–8932. 21266573 10.1074/jbc.M110.180638 PMC3058958

[102] Albanese F (2021) Constitutive silencing of LRRK2 kinase activity leads to early glucocerebrosidase deregulation and late impairment of autophagy *in vivo*. Neurobiology of Disease 159, 105487. 34419621 10.1016/j.nbd.2021.105487

[103] Zuo L (2021) 7,8-Dihydroxyflavone Ameliorates motor deficits via regulating autophagy in MPTP-induced mouse model of Parkinson's disease. Cell Death Discovery 7, 254. 34545064 10.1038/s41420-021-00643-5 PMC8452727

[104] Kang SY (2017) Autophagic modulation by rosuvastatin prevents rotenone-induced neurotoxicity in an *in vitro* model of Parkinson's disease. Neuroscience Letters 642, 20–26. 28137648 10.1016/j.neulet.2017.01.063

[105] Mammadova N (2019) Accelerated accumulation of retinal *α*-synuclein (pSer129) and tau, neuroinflammation, and autophagic dysregulation in a seeded mouse model of Parkinson's disease. Neurobiology of Disease 121, 1–16. 30218757 10.1016/j.nbd.2018.09.013

[106] Zhang Y (2019) Caffeic acid reduces A53T *α*-synuclein by activating JNK/Bcl-2-mediated autophagy in vitro and improves behaviour and protects dopaminergic neurons in a mouse model of Parkinson's disease. Pharmacological Research 150, 104538. 31707034 10.1016/j.phrs.2019.104538

[107] Corrochano S (2012) *α*-Synuclein levels affect autophagosome numbers *in vivo* and modulate Huntington disease pathology. Autophagy 8, 431–432. 22361581 10.4161/auto.19259

[108] Tomás-Zapico C (2012) *α*-Synuclein accumulates in huntingtin inclusions but forms independent filaments and its deficiency attenuates early phenotype in a mouse model of Huntington's disease. Human Molecular Genetics 21, 495–510. 22045698 10.1093/hmg/ddr507

[109] Ehrnhoefer DE (2018) Preventing mutant huntingtin proteolysis and intermittent fasting promote autophagy in models of Huntington disease. Acta Neuropathologica Communications 6, 16. 29510748 10.1186/s40478-018-0518-0 PMC5839066

[110] Fox LM (2020) Huntington's disease pathogenesis is modified *in vivo* by Alfy/Wdfy3 and selective macroautophagy. Neuron 105, 813–821, e6. 31899071 10.1016/j.neuron.2019.12.003 PMC7060123

[111] Fu H, Hardy J and Duff KE (2018) Selective vulnerability in neurodegenerative diseases. Nature Neuroscience 21, 1350–1358. 30250262 10.1038/s41593-018-0221-2 PMC6360529

[112] Hegde RN (2020) TBK1 phosphorylates mutant Huntingtin and suppresses its aggregation and toxicity in Huntington's disease models. The EMBO journal 39, e104671. 32757223 10.15252/embj.2020104671 PMC7459410

[113] Pinho BR (2020) The interplay between redox signaling and proteostasis in neurodegeneration: *in vivo* effects of a mitochondria-targeted antioxidant in Huntington's disease mice. Free Radical Biology & Medicine 146, 372–382. 31751762 10.1016/j.freeradbiomed.2019.11.021 PMC6970224

[114] Wold MS (2016) ULK1-mediated phosphorylation of ATG14 promotes autophagy and is impaired in Huntington's disease models. Molecular Neurodegeneration 11, 76. 27938392 10.1186/s13024-016-0141-0 PMC5148922

[115] Zhang Y, Sowers JR and Ren J (2018) Targeting autophagy in obesity: from pathophysiology to management. Nature reviews. Endocrinology 14, 356–376. 10.1038/s41574-018-0009-1 29686432

[116] Oga EA and Eseyin OR (2016) The obesity paradox and heart failure: a systematic review of a decade of evidence. Journal of Obesity 2016, 9040248. 26904277 10.1155/2016/9040248 PMC4745816

[117] Kovsan J (2011) Altered autophagy in human adipose tissues in obesity. The Journal of Clinical Endocrinology and Metabolism 96, E268–E277. 21047928 10.1210/jc.2010-1681

[118] Zhang X (2008) Hypothalamic IKKbeta/NF-kappaB and ER stress link overnutrition to energy imbalance and obesity. Cell 135, 61–73. 18854155 10.1016/j.cell.2008.07.043 PMC2586330

[119] Kaushik S (2012) Loss of autophagy in hypothalamic POMC neurons impairs lipolysis. EMBO reports 13, 258–265. 22249165 10.1038/embor.2011.260 PMC3323137

[120] Gonzalez CD (2011) The emerging role of autophagy in the pathophysiology of diabetes mellitus. Autophagy 7, 2–11. 20935516 10.4161/auto.7.1.13044 PMC3359481

[121] Santos RX (2014) Insulin therapy modulates mitochondrial dynamics and biogenesis, autophagy and tau protein phosphorylation in the brain of type 1 diabetic rats. Biochimica et Biophysica Acta 1842, 1154–1166. 24747740 10.1016/j.bbadis.2014.04.011

[122] Brännmark C (2013) Insulin signaling in type 2 diabetes: experimental and modeling analyses reveal mechanisms of insulin resistance in human adipocytes. The Journal of Biological Chemistry 288, 9867–9880. 23400783 10.1074/jbc.M112.432062 PMC3617287

[123] Ueno T and Komatsu M (2017) Autophagy in the liver: functions in health and disease. Nature Reviews Gastroenterology & Hepatology 14, 170–184. 28053338 10.1038/nrgastro.2016.185

[124] Huang SS (2017) Resveratrol protects podocytes against apoptosis via stimulation of autophagy in a mouse model of diabetic nephropathy. Scientific Reports 7, 45692. 28374806 10.1038/srep45692 PMC5379482

[125] Zheng J (2022) cPKC*γ* Deficiency exacerbates autophagy impairment and hyperphosphorylated Tau buildup through the AMPK/mTOR pathway in mice with type 1 diabetes mellitus. Neuroscience Bulletin 38, 1153–1169. 35596894 10.1007/s12264-022-00863-4 PMC9554100

[126] Jung HS (2008) Loss of autophagy diminishes pancreatic beta cell mass and function with resultant hyperglycemia. Cell Metabolism 8, 318–324. 18840362 10.1016/j.cmet.2008.08.013

[127] Ebato C (2008) Autophagy is important in islet homeostasis and compensatory increase of beta cell mass in response to high-fat diet. Cell Metabolism 8, 325–332. 18840363 10.1016/j.cmet.2008.08.009

[128] Jung HS and Lee MS (2009) Macroautophagy in homeostasis of pancreatic beta-cell. Autophagy 5, 241–243. 19066457 10.4161/auto.5.2.7518

[129] Marsh BJ (2007) Regulated autophagy controls hormone content in secretory-deficient pancreatic endocrine beta-cells. Molecular Endocrinology (Baltimore, Md.) 21, 2255–2269. 17579214 10.1210/me.2007-0077

[130] Quan W (2012) Autophagy deficiency in beta cells leads to compromised unfolded protein response and progression from obesity to diabetes in mice. Diabetologia 55, 392–403. 22075916 10.1007/s00125-011-2350-y

[131] Kaniuk NA (2007) Ubiquitinated-protein aggregates form in pancreatic beta-cells during diabetes-induced oxidative stress and are regulated by autophagy. Diabetes 56, 930–939. 17395740 10.2337/db06-1160

[132] Shigihara N (2014) Human IAPP-induced pancreatic *β* cell toxicity and its regulation by autophagy. The Journal of Clinical Investigation 124, 3634–3644. 25036706 10.1172/JCI69866 PMC4109539

[133] Jansen HJ (2012) Autophagy activity is up-regulated in adipose tissue of obese individuals and modulates proinflammatory cytokine expression. Endocrinology 153, 5866–5874. 23117929 10.1210/en.2012-1625

[134] Kosacka J (2015) Autophagy in adipose tissue of patients with obesity and type 2 diabetes. Molecular and Cellular Endocrinology 409, 21–32. 25818883 10.1016/j.mce.2015.03.015

[135] López-Vicario C (2015) Inhibition of soluble epoxide hydrolase modulates inflammation and autophagy in obese adipose tissue and liver: role for omega-3 epoxides. Proceedings of the National Academy of Sciences of the United States of America 112, 536–541. 25550510 10.1073/pnas.1422590112 PMC4299190

[136] Yan H, Gao Y and Zhang Y (2017) Inhibition of JNK suppresses autophagy and attenuates insulin resistance in a rat model of nonalcoholic fatty liver disease. Molecular Medicine Reports 15, 180–186. 27909723 10.3892/mmr.2016.5966 PMC5355648

[137] Chang E (2015) Ezetimibe improves hepatic steatosis in relation to autophagy in obese and diabetic rats. World Journal of Gastroenterology 21, 7754–7763. 26167075 10.3748/wjg.v21.i25.7754 PMC4491962

[138] Yang L (2010) Defective hepatic autophagy in obesity promotes ER stress and causes insulin resistance. Cell Metabolism 11, 467–478. 20519119 10.1016/j.cmet.2010.04.005 PMC2881480

[139] Komiya K (2010) Free fatty acids stimulate autophagy in pancreatic *β*-cells via JNK pathway. Biochemical and Biophysical Research Communications 401, 561–567. 20888798 10.1016/j.bbrc.2010.09.101

[140] He Q (2016) GLP-1 analogue improves hepatic lipid accumulation by inducing autophagy via AMPK/mTOR pathway. Biochemical and Biophysical Research Communications 476, 196–203. 27208776 10.1016/j.bbrc.2016.05.086

[141] Lai CH (2016) Multi-strain probiotics inhibit cardiac myopathies and autophagy to prevent heart injury in high-fat diet-fed rats. International Journal of Medical Sciences 13, 27–285. 10.7150/ijms.14769 PMC4829540 27076784

[142] Niso-Santano M (2019) Natural products in the promotion of healthspan and longevity. Clinical Pharmacology and Translational Medicine 3, 149. 31363716 10.31700/2572-7656.000123 PMC6666427

[143] Fu R (2018) A novel autophagy inhibitor berbamine blocks SNARE-mediated autophagosome-lysosome fusion through upregulation of BNIP3. Cell Death & Disease 9, 1–15. 29445175 10.1038/s41419-018-0276-8 PMC5833711

[144] Tang ZH (2018) Identification of a novel autophagic inhibitor cepharanthine to enhance the anti-cancer property of dacomitinib in non-small cell lung cancer. Cancer Letters 412, 1–9. 29024815 10.1016/j.canlet.2017.10.001

[145] Wu MY (2017) Natural autophagy blockers, dauricine (DAC) and daurisoline (DAS), sensitize cancer cells to camptothecin-induced toxicity. Oncotarget 8, 77673. 29100416 10.18632/oncotarget.20767 PMC5652807

[146] Zhou J (2015) A novel autophagy/mitophagy inhibitor liensinine sensitizes breast cancer cells to chemotherapy through DNM1L-mediated mitochondrial fission. Autophagy 11, 1259–1279. 26114658 10.1080/15548627.2015.1056970 PMC4590597

[147] Xie SB, He XX and Yao SK (2015) Matrine-induced autophagy regulated by p53 through AMP-activated protein kinase in human hepatoma cells. International Journal of Oncology 47, 517–526. 26034977 10.3892/ijo.2015.3023

[148] Song L (2017) Natural cyclopeptide RA-XII, a new autophagy inhibitor, suppresses protective autophagy for enhancing apoptosis through AMPK/mTOR/P70S6K pathways in HepG2 cells. Molecules 22, 1934. 29137114 10.3390/molecules22111934 PMC6150396

[149] Cui Q (2007) Oridonin induced autophagy in human cervical carcinoma HeLa cells through Ras, JNK, and P38 regulation. Journal of Pharmacological Sciences 105, 317–325. 18094523 10.1254/jphs.fp0070336

[150] Roy B (2014) Role of PI3K/Akt/mTOR and MEK/ERK pathway in Concanavalin A induced autophagy in HeLa cells. Chemico-Biological Interactions 210, 96–102. 24434245 10.1016/j.cbi.2014.01.003

[151] Gao J (2018) The anticancer effects of ferulic acid is associated with induction of cell cycle arrest and autophagy in cervical cancer cells. Cancer Cell International 18, 1–9. 30013454 10.1186/s12935-018-0595-y PMC6045836

[152] Kim TW (2018) Kaempferol induces autophagic cell death via IRE1-JNK-CHOP pathway and inhibition of G9a in gastric cancer cells. Cell Death & Disease 9, 1–14. 30158521 10.1038/s41419-018-0930-1 PMC6115440

[153] Lan CY (2019) Quercetin facilitates cell death and chemosensitivity through RAGE/PI3K/AKT/mTOR axis in human pancreatic cancer cells. Journal of Food and Drug Analysis 27, 887–896. 31590760 10.1016/j.jfda.2019.07.001 PMC9306979

[154] Ye Y (2016) 3, 3′-Diindolylmethane induces anti-human gastric cancer cells by the miR-30e-ATG5 modulating autophagy. Biochemical Pharmacology 115, 77–84. 27372603 10.1016/j.bcp.2016.06.018

[155] Kaushik G (2015) Honokiol inhibits melanoma stem cells by targeting notch signaling. Molecular Carcinogenesis 54, 1710–1721. 25491779 10.1002/mc.22242 PMC4776032

[156] Park KR (2022) Paeoniflorigenone regulates apoptosis, autophagy, and necroptosis to induce anti-cancer bioactivities in human head and neck squamous cell carcinomas. Journal of Ethnopharmacology 288, 115000. 35051602 10.1016/j.jep.2022.115000

[157] Park EJ, Choi KS and Kwon TK (2011) *β*-Lapachone-induced reactive oxygen species (ROS) generation mediates autophagic cell death in glioma U87 MG cells. Chemico-Biological Interactions 189, 37–44. 21035436 10.1016/j.cbi.2010.10.013

[158] Li YC (2014) Plumbagin induces apoptotic and autophagic cell death through inhibition of the PI3K/Akt/mTOR pathway in human non-small cell lung cancer cells. Cancer Letters 344, 239–259. 24280585 10.1016/j.canlet.2013.11.001

[159] Shailasree S (2015) Cytotoxic effect of p-coumaric acid on neuroblastoma, N2a cell via generation of reactive oxygen species leading to dysfunction of mitochondria inducing apoptosis and autophagy. Molecular Neurobiology 51, 119–130. 24760364 10.1007/s12035-014-8700-2

[160] Wang G (2017) The novel autophagy inhibitor elaiophylin exerts antitumor activity against multiple myeloma with mutant TP53 in part through endoplasmic reticulum stress-induced apoptosis. Cancer Biology & Therapy 18, 584–595. 28718729 10.1080/15384047.2017.1345386 PMC5653199

[161] Aoki H (2007) Evidence that curcumin suppresses the growth of malignant gliomas in vitro and in vivo through induction of autophagy: role of Akt and extracellular signal-regulated kinase signaling pathways. Molecular Pharmacology 72, 29–39. 17395690 10.1124/mol.106.033167

[162] Teiten MH (2011) Anti-proliferative potential of curcumin in androgen-dependent prostate cancer cells occurs through modulation of the wingless signaling pathway. International Journal of Oncology 38, 603–611. 21240460 10.3892/ijo.2011.905

[163] Lee YJ (2011) Involvement of ROS in curcumin-induced autophagic cell death. The Korean Journal of Physiology & Pharmacology 15, 1–7. 21461234 10.4196/kjpp.2011.15.1.1 PMC3062078

[164] Kim JY (2012) Curcumin-induced autophagy contributes to the decreased survival of oral cancer cells. Archives of Oral Biology 57, 1018–1025. 22554995 10.1016/j.archoralbio.2012.04.005

[165] Zhu Y and Bu S (2017) Curcumin induces autophagy, apoptosis, and cell cycle arrest in human pancreatic cancer cells. Evidence-Based Complementary and Alternative Medicine 2017, 5787218. 29081818 10.1155/2017/5787218 PMC5610853

[166] Liu J (2017) Curcumin sensitizes prostate cancer cells to radiation partly via epigenetic activation of miR-143 and miR-143 mediated autophagy inhibition. Journal of Drug Targeting 25, 645–652. 28391715 10.1080/1061186X.2017.1315686

[167] Muniraj N (2019) Withaferin A inhibits lysosomal activity to block autophagic flux and induces apoptosis via energetic impairment in breast cancer cells. Carcinogenesis 40, 1110–1120. 10.1093/carcin/bgz015 PMC10893887 30698683

[168] Zhang H (2022) Periplocin induces apoptosis of pancreatic cancer cells through autophagy via the AMPK/mTOR pathway. Journal of Oncology 2022, 8055004. 35847371 10.1155/2022/8055004 PMC9277210

[169] Tian Y (2019) Resveratrol as a natural regulator of autophagy for prevention and treatment of cancer. OncoTargets and Therapy 17, 8601–8609. 10.2147/OTT.S213043 PMC6802539 31802896

[170] Fu Y (2014) Resveratrol inhibits breast cancer stem-like cells and induces autophagy via suppressing Wnt/*β*-catenin signaling pathway. PLoS ONE 9, e102535. 25068516 10.1371/journal.pone.0102535 PMC4113212

[171] Miki H (2012) Resveratrol induces apoptosis via ROS-triggered autophagy in human colon cancer cells. International Journal of Oncology 40, 1020–1028. 22218562 10.3892/ijo.2012.1325 PMC3584586

[172] Ferraresi A (2017) Resveratrol inhibits IL-6-induced ovarian cancer cell migration through epigenetic up-regulation of autophagy. Molecular Carcinogenesis 56, 1164–1181. 27787915 10.1002/mc.22582

[173] Xu J (2019) Corilagin induces apoptosis, autophagy and ROS generation in gastric cancer cells *in vitro*. International Journal of Molecular Medicine 43, 967–979. 30569134 10.3892/ijmm.2018.4031 PMC6317684

[174] Wang SG (2013) Punicalagin induces apoptotic and autophagic cell death in human U87MG glioma cells. Acta Pharmacologica Sinica 34, 1411–1419. 24077634 10.1038/aps.2013.98 PMC4006469

[175] Chen SY (2022) Lucidone inhibits autophagy and MDR1 via HMGB1/RAGE/PI3K/Akt signaling pathway in pancreatic cancer cells. Phytotherapy Research 36, 1664–1677. 35224793 10.1002/ptr.7385

[176] Guo J (2015) Celastrol induces autophagy by targeting AR/miR-101 in prostate cancer cells. PLoS ONE 10, e0140745. 26473737 10.1371/journal.pone.0140745 PMC4608724

[177] Zhang J (2022) Toosendanin and isotoosendanin suppress triple-negative breast cancer growth via inducing necrosis, apoptosis and autophagy. Chemico-Biological Interactions 351, 109739. 34742683 10.1016/j.cbi.2021.109739

[178] Wu X (2021) Autophagy and cardiac diseases: therapeutic potential of natural products. Medicinal Research Reviews 41, 314–341. 32969064 10.1002/med.21733

[179] Huang Z (2015) Berberine alleviates cardiac ischemia/reperfusion injury by inhibiting excessive autophagy in cardiomyocytes. European Journal of Pharmacology 762, 1–10. 26004523 10.1016/j.ejphar.2015.05.028

[180] Zeng Z (2019) Myocardial hypertrophy is improved with berberine treatment via long non-coding RNA MIAT-mediated autophagy. Journal of Pharmacy and Pharmacology 71, 1822–1831. 31612504 10.1111/jphp.13170

[181] Wang X (2019) Tanshinone IIA restores dynamic balance of autophagosome/autolysosome in doxorubicin-induced cardiotoxicity via targeting beclin1/LAMP1. Cancers 11, 910. 31261758 10.3390/cancers11070910 PMC6679133

[182] Xu M (2019) Oridonin protects against cardiac hypertrophy by promoting P21-related autophagy. Cell Death & Disease 10, 1–16. 10.1038/s41419-019-1617-y PMC6534559 31127082

[183] Li X (2018) Inhibition of autophagy via activation of PI3K/Akt/mTOR pathway contributes to the protection of hesperidin against myocardial ischemia/reperfusion injury. International Journal of Molecular Medicine 42, 1917–1924. 30066841 10.3892/ijmm.2018.3794 PMC6108872

[184] Hu L (2022) Icariin inhibits isoproterenol-induced cardiomyocyte hypertropic injury through activating autophagy via the AMPK/mTOR signaling pathway. Biochemical and Biophysical Research Communications 593, 65–72. 35063771 10.1016/j.bbrc.2022.01.029

[185] Hu J (2016) Luteolin alleviates post-infarction cardiac dysfunction by up-regulating autophagy through Mst1 inhibition. Journal of Cellular and Molecular Medicine 20, 147–156. 26538370 10.1111/jcmm.12714 PMC4717847

[186] Wu X (2017) Nobiletin attenuates adverse cardiac remodeling after acute myocardial infarction in rats via restoring autophagy flux. Biochemical and Biophysical Research Communications 492, 262–268. 28830813 10.1016/j.bbrc.2017.08.064

[187] Liu B (2015) Puerarin prevents cardiac hypertrophy induced by pressure overload through activation of autophagy. Biochemical and Biophysical Research Communications 464, 908–915. 26188094 10.1016/j.bbrc.2015.07.065

[188] Chen WR (2018) Melatonin attenuates myocardial ischemia/reperfusion injury by inhibiting autophagy via an AMPK/mTOR signaling pathway. *Cellular* *Physiology and Biochemistry :* International Journal of Experimental Cellular Physiology, Biochemistry, and Pharmacology 47, 2067–2076. 10.1159/000491474 29975938

[189] Xie S (2015) Melatonin protects against chronic intermittent hypoxia-induced cardiac hypertrophy by modulating autophagy through the 5′ adenosine monophosphate-activated protein kinase pathway. Biochemical and Biophysical Research Communications 464, 975–981. 26188509 10.1016/j.bbrc.2015.06.149

[190] Xiao H (2022) Hinokitiol protects cardiomyocyte from oxidative damage by inhibiting GSK3*β*-mediated autophagy. Oxidative Medicine and Cellular Longevity 2022, 1–13. 10.1155/2022/2700000 PMC9001072 35419165

[191] Liu H (2019) Role of thymoquinone in cardiac damage caused by sepsis from BALB/c mice. Inflammation 42, 516–525. 30343389 10.1007/s10753-018-0909-1

[192] Yan X (2019) Gallic acid suppresses cardiac hypertrophic remodeling and heart failure. Molecular Nutrition & Food Research 63, 1800807. 10.1002/mnfr.201800807 30521107

[193] Eisenberg T (2016) Cardioprotection and lifespan extension by the natural polyamine spermidine. Nature Medicine 22, 1428–1438. 10.1038/nm.4222 PMC5806691 27841876

[194] Yan J (2019) Spermidine-enhanced autophagic flux improves cardiac dysfunction following myocardial infarction by targeting the AMPK/mTOR signalling pathway. British Journal of Pharmacology 176, 3126–3142. 31077347 10.1111/bph.14706 PMC6692641

[195] Liu R (2018) Curcumin alleviates isoproterenol-induced cardiac hypertrophy and fibrosis through inhibition of autophagy and activation of mTOR. European Review for Medical and Pharmacological Sciences 22, 7500–7508. 30468499 10.26355/eurrev_201811_16291

[196] Ba L (2019) Allicin attenuates pathological cardiac hypertrophy by inhibiting autophagy via activation of PI3K/Akt/mTOR and MAPK/ERK/mTOR signaling pathways. Phytomedicine: International Journal of Phytotherapy and Phytopharmacology 58, 152765. 31005720 10.1016/j.phymed.2018.11.025

[197] Gu J (2018) Resveratrol suppresses doxorubicin-induced cardiotoxicity by disrupting E2F1 mediated autophagy inhibition and apoptosis promotion. Biochemical Pharmacology 150, 202–213. 29475062 10.1016/j.bcp.2018.02.025

[198] Xu X (2012) Resveratrol attenuates doxorubicin-induced cardiomyocyte death via inhibition of p70 S6 kinase 1-mediated autophagy. Journal of Pharmacology and Experimental Therapeutics 341, 183–195. 22209892 10.1124/jpet.111.189589 PMC3310694

[199] Sun GZ (2020) Ginsenoside Rg3 protects heart against isoproterenol-induced myocardial infarction by activating AMPK mediated autophagy. Cardiovascular Diagnosis and Therapy 10, 153–160. 32420095 10.21037/cdt.2020.01.02 PMC7225426

[200] Xiao Y (2017) Cucurbitacin B protects against pressure overload induced cardiac hypertrophy. Journal of Cellular Biochemistry 118, 3899–3910. 28390176 10.1002/jcb.26041

[201] Huang M (2017) Berberine improves cognitive impairment by promoting autophagic clearance and inhibiting production of *β*-amyloid in APP/tau/PS1 mouse model of Alzheimer's disease. Experimental Gerontology 91, 25–33. 28223223 10.1016/j.exger.2017.02.004

[202] Umezawa K (2018) Therapeutic activity of plant-derived alkaloid conophylline on metabolic syndrome and neurodegenerative disease models. Human Cell 31, 95–101. 29249016 10.1007/s13577-017-0196-4

[203] Wang C (2014) Downregulation of PI3K/Akt/mTOR signaling pathway in curcumin-induced autophagy in APP/PS1 double transgenic mice. European Journal of Pharmacology 740, 312–320. 25041840 10.1016/j.ejphar.2014.06.051

[204] Fan Y (2017) Identification of natural products with neuronal and metabolic benefits through autophagy induction. Autophagy 13, 41–56. 27791467 10.1080/15548627.2016.1240855 PMC5240827

[205] Yang C (2022) Celastrol enhances transcription factor EB (TFEB)-mediated autophagy and mitigates Tau pathology: implications for Alzheimer's disease therapy. Acta Pharmaceutica Sinica B 12, 1707–1722. 35847498 10.1016/j.apsb.2022.01.017 PMC9279716

[206] Moon J (2014) Caffeine prevents human prion-protein-mediated neurotoxicity through the induction of autophagy. International Journal of Molecular Medicine 34, 553–558. 24938171 10.3892/ijmm.2014.1814

[207] Li LS (2017) *Dendrobium nobile* Lindl alkaloid, a novel autophagy inducer, protects against axonal degeneration induced by A*β*25-35 in hippocampus neurons *in vitro*. CNS Neuroscience & Therapeutics 23, 329–340. 28261990 10.1111/cns.12678 PMC6492701

[208] Liang J (2019) Enhancing the retrograde axonal transport by curcumin promotes autophagic flux in N2a/APP695swe cells. Aging (Albany NY 11, 7036–7050. 31488728 10.18632/aging.102235 PMC6756876

[209] Zhang L (2018) The potential protective effect of curcumin on amyloid-*β*-42 induced cytotoxicity in HT-22 cells. BioMed Research International 2018, 8134902. 29568765 10.1155/2018/8134902 PMC5820551

[210] Wang N (2020) *β*-Asarone inhibits amyloid-*β* by promoting autophagy in a cell model of Alzheimer's disease. Frontiers in Pharmacology 10, 1529. 32009952 10.3389/fphar.2019.01529 PMC6979317

[211] Li X, Song J and Dong R (2019) Cubeben induces autophagy via PI3K-AKT-mTOR pathway to protect primary neurons against amyloid beta in Alzheimer's disease. Cytotechnology 71, 679–686. 30968233 10.1007/s10616-019-00313-6 PMC6546769

[212] Zhu Y and Wang J (2015) Wogonin increases *β*-amyloid clearance and inhibits tau phosphorylation via inhibition of mammalian target of rapamycin: potential drug to treat Alzheimer's disease. Neurological Sciences 36, 1181–1188. 25596147 10.1007/s10072-015-2070-z

[213] Kuang L, Cao X and Lu Z (2017) Baicalein protects against rotenone-induced neurotoxicity through induction of autophagy. Biological and Pharmaceutical Bulletin 40, 1537–1543. 28659545 10.1248/bpb.b17-00392

[214] Chaouhan HS (2022) Calycosin alleviates paraquat-induced neurodegeneration by improving mitochondrial functions and regulating autophagy in a drosophila model of Parkinson's disease. Antioxidants 11, 222. 35204105 10.3390/antiox11020222 PMC8868496

[215] El-Horany HE (2016) Ameliorative effect of quercetin on neurochemical and behavioral deficits in rotenone rat model of Parkinson's disease: modulating autophagy (quercetin on experimental Parkinson's disease). Journal of Biochemical and Molecular Toxicology 30, 360–369. 27252111 10.1002/jbt.21821

[216] Hsueh KW (2016) Autophagic down-regulation in motor neurons remarkably prolongs the survival of ALS mice. Neuropharmacology 108, 152–160. 27059126 10.1016/j.neuropharm.2016.03.035

[217] Achour I (2016) Oleuropein prevents neuronal death, mitigates mitochondrial superoxide production and modulates autophagy in a dopaminergic cellular model. International Journal of Molecular Sciences 17, 1293. 27517912 10.3390/ijms17081293 PMC5000690

[218] Rigacci S (2015) Oleuropein aglycone induces autophagy via the AMPK/mTOR signalling pathway: a mechanistic insight. Oncotarget 6, 35344. 26474288 10.18632/oncotarget.6119 PMC4742109

[219] Vidoni C (2018) Resveratrol protects neuronal-like cells expressing mutant Huntingtin from dopamine toxicity by rescuing ATG4-mediated autophagosome formation. Neurochemistry International 117, 174–187. 28532681 10.1016/j.neuint.2017.05.013

[220] Lin TK (2014) Resveratrol partially prevents rotenone-induced neurotoxicity in dopaminergic SH-SY5Y cells through induction of heme oxygenase-1 dependent autophagy. International Journal of Molecular Sciences 15, 1625–1646. 24451142 10.3390/ijms15011625 PMC3907890

[221] Deng H and Mi MT (2016) Resveratrol attenuates A*β*25–35 caused neurotoxicity by inducing autophagy through the TyrRS-PARP1-SIRT1 signaling pathway. Neurochemical Research 41, 2367–2379. 27180189 10.1007/s11064-016-1950-9

[222] Guo YJ (2016) Resveratrol alleviates MPTP-induced motor impairments and pathological changes by autophagic degradation of *α*-synuclein via SIRT1-deacetylated LC3. Molecular Nutrition & Food Research 60, 2161–2175. 27296520 10.1002/mnfr.201600111 PMC6089356

[223] Russo R (2014) Role of D-Limonene in autophagy induced by bergamot essential oil in SH-SY5Y neuroblastoma cells. PLoS ONE 9, e113682. 25419658 10.1371/journal.pone.0113682 PMC4242674

[224] Arel-Dubeau AM (2014) Cucurbitacin E has neuroprotective properties and autophagic modulating activities on dopaminergic neurons. Oxidative Medicine and Cellular Longevity 2014, 1–15. 10.1155/2014/425496 PMC4276330 25574337

[225] Rekha K and Sivakamasundari R (2018) Geraniol protects against the protein and oxidative stress induced by rotenone in an in vitro model of Parkinson's disease. Neurochemical Research 43, 1947–1962. 30141137 10.1007/s11064-018-2617-5

[226] Wang Z (2022) Cannabidiol induces autophagy and improves neuronal health associated with SIRT1 mediated longevity. GeroScience 44, 1505–1524. 35445360 10.1007/s11357-022-00559-7 PMC9213613

[227] Deng Y (2014) Berberine attenuates autophagy in adipocytes by targeting BECN1. Autophagy 10, 1776–1786. 25126729 10.4161/auto.29746 PMC4198362

[228] Baur JA (2006) Resveratrol improves health and survival of mice on a high-calorie diet. Nature 444, 337–342. 17086191 10.1038/nature05354 PMC4990206

[229] Wang B (2014) Resveratrol-enhanced autophagic flux ameliorates myocardial oxidative stress injury in diabetic mice. Journal of Cellular and Molecular Medicine 18, 1599–1611. 24889822 10.1111/jcmm.12312 PMC4190906

[230] Qu X (2019) Resveratrol alleviates ischemia/reperfusion injury of diabetic myocardium via inducing autophagy. Experimental and Therapeutic Medicine 18, 2719–2725. 31555372 10.3892/etm.2019.7846 PMC6755413

[231] Yan J (2012) Enhanced autophagy plays a cardinal role in mitochondrial dysfunction in type 2 diabetic Goto–Kakizaki (GK) rats: ameliorating effects of (−)-epigallocatechin-3-gallate. The Journal of Nutritional Biochemistry 23, 716–724. 21820301 10.1016/j.jnutbio.2011.03.014

[232] Xu C (2019) Trehalose restores functional autophagy suppressed by high glucose. Reproductive Toxicology 85, 51–58. 30769031 10.1016/j.reprotox.2019.02.005 PMC7271258

[233] Shi L (2015) Dihydromyricetin improves skeletal muscle insulin sensitivity by inducing autophagy via the AMPK-PGC-1*α*-Sirt3 signaling pathway. Endocrine 50, 378–389. 25896550 10.1007/s12020-015-0599-5

[234] Xu X (2020) The effects of puerarin on autophagy through regulating of the PERK/eIF2*α*/ATF4 signaling pathway influences renal function in diabetic nephropathy. Diabetes, Metabolic Syndrome and Obesity: Targets and Therapy 13, 2583–2592. 32765037 10.2147/DMSO.S256457 PMC7381766

[235] Zhang M (2017) Melatonin protects against diabetic cardiomyopathy through Mst1/Sirt3 signaling. Journal of Pineal Research 63, e12418. 10.1111/jpi.12418 28480597

[236] Ma R (2022) Ferulic acid ameliorates renal injury via improving autophagy to inhibit inflammation in diabetic nephropathy mice. Biomedicine & Pharmacotherapy 153, 113424. 36076545 10.1016/j.biopha.2022.113424

[237] Zhang XX (2022) Arjunolic acid from Cyclocarya paliurus ameliorates diabetic retinopathy through AMPK/mTOR/HO-1 regulated autophagy pathway. Journal of Ethnopharmacology 284, 114772. 34688801 10.1016/j.jep.2021.114772

[238] Yao Q (2018) Curcumin protects against diabetic cardiomyopathy by promoting autophagy and alleviating apoptosis. Journal of Molecular and Cellular Cardiology 124, 26–34. 30292723 10.1016/j.yjmcc.2018.10.004

[239] Zhang P (2020) Curcumin inhibited podocyte cell apoptosis and accelerated cell autophagy in diabetic nephropathy via regulating Beclin1/UVRAG/Bcl2. Diabetes, Metabolic Syndrome and Obesity: Targets and Therapy 13, 641. 32184643 10.2147/DMSO.S237451 PMC7060797

